# Genotypic differences between strains of the opportunistic pathogen *Corynebacterium bovis* isolated from humans, cows, and rodents

**DOI:** 10.1371/journal.pone.0209231

**Published:** 2018-12-26

**Authors:** Christopher Cheleuitte-Nieves, Christopher A. Gulvik, John R. McQuiston, Ben W. Humrighouse, Melissa E. Bell, Aaron Villarma, Vincent A. Fischetti, Lars F. Westblade, Neil S. Lipman

**Affiliations:** 1 Tri-Institutional Training Program in Laboratory Animal Medicine and Science, Memorial Sloan Kettering Cancer Center, Weill Cornell Medicine, and The Rockefeller University, New York, New York, United States of America; 2 Center of Comparative Medicine and Pathology, Memorial Sloan Kettering Cancer Center and Weill Cornell Medicine, New York, New York, United States of America; 3 Special Bacteriology Reference Laboratory, Bacterial Special Pathogens Branch, Division of High-Consequence Pathogens and Pathology, Centers for Disease Control and Prevention, Atlanta, Georgia, United States of America; 4 Laboratory of Bacterial Pathogenesis and Immunology, The Rockefeller University, New York, New York, United States of America; 5 Department of Pathology and Laboratory Medicine, Weill Cornell Medicine, New York, New York, United States of America; 6 Department of Medicine, Division of Infectious Diseases, Weill Cornell Medicine, New York, New York, United States of America; Tianjin University, CHINA

## Abstract

*Corynebacterium bovis* is an opportunistic bacterial pathogen shown to cause eye and prosthetic joint infections as well as abscesses in humans, mastitis in dairy cattle, and skin disease in laboratory mice and rats. Little is known about the genetic characteristics and genomic diversity of *C*. *bovis* because only a single draft genome is available for the species. The overall aim of this study was to sequence and compare the genome of *C*. *bovis* isolates obtained from different species, locations, and time points. Whole-genome sequencing was conducted on 20 *C*. *bovis* isolates (six human, four bovine, nine mouse and one rat) using the Illumina MiSeq platform and submitted to various comparative analysis tools. Sequencing generated high-quality contigs (over 2.53 Mbp) that were comparable to the only reported assembly using *C*. *bovis* DSM 20582^T^ (97.8 ± 0.36% completeness). The number of protein-coding DNA sequences (2,174 ± 12.4) was similar among all isolates. A *Corynebacterium* genus neighbor-joining tree was created, which revealed *Corynebacterium falsenii* as the nearest neighbor to *C*. *bovis* (95.87% similarity), although the reciprocal comparison shows *Corynebacterium jeikeium* as closest neighbor to *C*. *falsenii*. Interestingly, the average nucleotide identity demonstrated that the *C*. *bovis* isolates clustered by host, with human and bovine isolates clustering together, and the mouse and rat isolates forming a separate group. The average number of genomic islands and putative virulence factors were significantly higher (p<0.001) in the mouse and rat isolates as compared to human/bovine isolates. *Corynebacterium bovis’* pan-genome contained a total of 3,067 genes of which 1,354 represented core genes. The known core genes of all isolates were primarily related to ‘‘metabolism” and ‘‘information storage/processing.” However, most genes were classified as ‘‘function unknown” or “unclassified”. Surprisingly, no intact prophages were found in any isolate; however, almost all isolates had at least one complete CRISPR-Cas system.

## Introduction

*Corynebacterium bovis* is a small Gram-positive bacterial rod belonging to the family *Corynebacteriaceae* that has been reported to be an opportunistic pathogen in several species including humans [1–6]. Recently, a series of nine clinical cases of *C*. *bovis* infection in humans was reported [3]. These cases included infections of the eye, cysts and prosthetic joints. Furthermore, in 2016 and 2017, four *C*. *bovis* isolates were isolated from human subjects in a single health care institution in New York City, USA (this work). *Corynebacterium bovis* is the most frequently isolated *Corynebacterium* species from intramammary infections in dairy cows and has been associated with reduced milk production and mastitis [3, 7–9]. Mastitis in dairy cows is an economically important disease in which ~30% of lactating cows on New York State dairy farms, 19.8% of dairy herds in Ontario, Canada, and up to 52.7% of Estonian dairy herds are affected [8]. In 1998, the causative agent of ‘scaly skin disease’ in immunocompromised nude mice, more recently referred to as *Corynebacterium*-associated hyperkeratosis (CAH), was identified as *C*. *bovis* via 16S rRNA gene sequence analysis [10]. In mice, *C*. *bovis* colonizes the superficial layers of the epidermis of immunocompromised mice causing a highly contagious, severe, orthokeratotic hyperkeratosis and acanthosis [1, 5, 10]. The use and availability of a wide array of immunocompromised mouse models has increased exponentially over the past decade as they serve as valuable tools in oncology and immunology, as well as in other scientific disciplines, supporting the growth of xenografts and allografts [11, 12]. *C*. *bovis* is thought to have delayed or slowed tumor development leading to failed engraftment [13]. The associated physiologic and immunologic complications can be profound making infected animals unsuitable for some research use [4, 5, 14].

At present, little is known about the molecular characteristics of *C*. *bovis* and the genomic diversity among different isolates. Brooks and Barnum, (1984) studied the biochemical reactions and morphological characteristics of various bovine and human isolates [7]. These authors found highly variable biochemical results among the isolates suggesting that multiple phenotypes occur within the species. They also highlighted the need to perform genomic analysis to reliably characterize and compare *C*. *bovis* isolates and more accurately determine if different strains exist, which remains unknown. A previous study investigated the pathogenicity and genetic variation of three *C*. *bovis* isolates in immunodeficient mice using a 16S rRNA gene-based assay [15]. They compared a hyperkeratosis-associated isolate, an isolate from asymptomatic colonized nude mice and the Type strain (DSM 20582^T^; ATCC 7715^T^) of bovine origin, and found nucleotide and biochemical differences between strains. However there were no differences observed in the growth, transmission, incidence, nor severity of hyperkeratosis or acanthosis, following experimental infection of 37 mice. Another study compared four *C*. *bovis* isolates obtained from human eye infections with the Type strain (ATCC 7715^T^, bovine origin) using 16S rRNA gene sequence analysis and found that they were identical [3]. However, these authors emphasized that human and animal strains do vary in their biochemical properties and 16S rRNA gene sequence analysis lacks the precision and accuracy offered by whole-genome sequencing (WGS) [16]. WGS (using “next-generation” DNA sequencing technologies) has allowed the broad examination of the genomic content and population structure of bacterial species [17]. The use of comparative genomics facilitates the differentiation of bacteria at the molecular level allowing for the characterization of the pangenome, i.e., the entire gene set of all strains of a given species, and the evolutionary relationships among related species [18]. For example, previous work used WGS to identify significant differences in the composition of pathogenicity islands among *Corynebacterium pseudotuberculosis ovis* and *equi* biovars and demonstrated clonal behavior, i.e., different genomes with similar genetic content, among strains that infect small ruminants (biovar *ovis*) and greater plasticity, i.e., gene variability among strains, in strains belonging to the biovar *equi* [19].

Currently, there is a single draft *C*. *bovis* genome sequence, which is the species’ Type strain DSM 20582^T^ (= ATCC 7715^T^) from an isolate cultured from a bovine udder [8]. The genome is 2.52 Mbp and encodes 2,339 predicted proteins. However, the question remains whether there are significant genomic differences between isolates obtained from different host species. WGS allows for a comprehensive exploration of the genomic content, population structure, and diversity of different *C*. *bovis* isolates to be conducted [17]. Understanding the molecular characteristics of *C*. *bovis* will permit discrimination of subtle genetic differences and exploration of differences in pathogenicity characteristics.

The aim of this study was to sequence, characterize, and compare the genomes of 20 *C*. *bovis* isolates obtained from four distinct host species, five geographic locations, and five time points from 1959 through 2017, and compare them to the already sequenced species Type strain. We hypothesized that genomic differences would be observed and these differences may reveal unique characteristics, which could aid in understanding epidemiologic relationships among isolates as well as whether or not there is strain specificity among hosts. Comparative genomic analyses, which included major genomic features; genomic similarities and differences among isolates; determination of the pan- and core genomes, and singleton composition; characterization of functional gene categories using the cluster of orthologous genes (COGs) classification; and, prediction of genomic islands, virulence factors, prophages, and CRISPR-Cas (Clustered Regularly Interspaced Short Palindromic Repeats/CRISPR-associated) systems, were performed.

## Methods

### Isolate collection and cultivation

This study did not require committee approvals. The *C*. *bovis* isolates were obtained from humans, cattle, mice, and a rat (Table 1). Samples were isolated from clinically affected humans or animals at the Memorial Sloan Kettering Cancer Center (MSK), New York, NY; NewYork-Presbyterian Hospital-Weill Cornell Medical Center (NYPH-WCMC), New York, NY; Weill Cornell Medicine (WCM), New York, NY; University of Colorado (UC), Denver, CO; University of Tennessee (UT), Knoxville, TN; Mispro Biotech Services, New York, NY; and the Centers for Disease Control and Prevention (CDC)—Special Bacteriology Reference Laboratory (SBRL) repository. The associated clinical signs included wounds in humans, mastitis in cattle, skin disease (hyperkeratosis and acanthosis) in mice, and dermatitis with ulcerative lesions on the limbs of a rat.

Isolates that had been previously confirmed, using colorimetric biochemical testing (API Coryne, bioMérieux, Marcy l’Etoile, France) and/or using matrix-assisted laser desorption/ionization time-of-flight mass spectrometry (Bruker MALDI-TOF Biotyper system, IDEXX BioResearch Microbiology Services, Columbia, MO), as *C*. *bovis* were collated and cultured on tryptic soy agar supplemented with soy lecithin and polysorbate 80 (prepared in-house at the CDC) and incubated at 37°C for 48 h in preparation for WGS. *Corynebacterium bovis* has been reported to exhibit two colonial phenotypes, small (~1 mm diameter) and wild-type large (~2 mm diameter), after subculturing [1]. The small colony-type appears to be less stable than the large wild-type as further subculturing of the small colony type yielded a mixed population of small and large (wild-type) colony types in previous studies. The small colony types were originally observed in four of our isolates (one obtained from a human and three from mice) and were maintained separate for independent sequencing.

**Table 1:** *Corynebacterium bovis* isolates evaluated in this study.

Host	Isolate Identifier	Origin of Isolate	Institution	GenBank accession N°
Bovine (milk/mastitis)	DSM 20582^T^	Germany (2012)	GenBank (ATCC 7715)	AENJ00000000
Bovine (milk/mastitis)	4826	Hawaii, USA (1959)	CDC SBRL	PQNX00000000
Bovine (milk/mastitis)	4828	Hawaii, USA (1959)	CDC SBRL	PQNW00000000
Bovine (milk/mastitis)	MI 82–1021	Tennessee, USA (1982)	UT	PQNJ00000000
Human, female	F6900	Washington, USA (1985)	CDC SBRL	PQNK00000000
Human, female	WCM1	New York, USA (2016)	NYPH-WCMC	PQNI00000000
Human, female	WCM3-LARGE^a^	New York, USA (2016)	NYPH-WCMC	PQNH00000000
Human, female	WCM3-SMALL^a^	New York, USA (2016)	NYPH-WCMC	PQNG00000000
Human, female	WCM4	New York, USA (2017)	NYPH-WCMC	PQNF00000000
Human, female	WCM5	New York, USA (2017)	NYPH-WCMC	PQNE00000000
Mouse (Athymic nude, skin)	CUAMC1-LARGE^a^	Colorado,USA (2014)	UC-Anschutz Medical Campus	PQNM00000000
Mouse (Athymic nude, skin)	CUAMC1-SMALL^a^	Colorado, USA (2014)	UC-Anschutz Medical Campus	PQNL00000000
Mouse (Athymic nude, skin)	7894	New York, USA (2008)	MSK	PQNV00000000
Mouse (Athymic nude, skin)	16-1683-LARGE^a^	New York, USA (2016)	WCM	PQNS00000000
Mouse (Athymic nude, skin)	16-1683-SMALL^a^	New York, USA (2016)	WCM	PQNR00000000
Mouse (Athymic nude, skin)	17-0240-LARGE^a^	New York, USA (2017)	MSK	PQNO00000000
Mouse (Athymic nude, skin)	17-0240-SMALL^a^	New York, USA (2017)	MSK	PQNN00000000
Mouse (NSG, skin)^b^	16–3465	New York, USA (2016)	WCM	PQNP00000000
Mouse (NSG, skin)^b^	13–1426	New York, USA (2013)	Mispro Biotech Services	PQNT00000000
Mouse (Athymic nude, skin)	12–5346	New York, USA (2012)	Mispro Biotech Services	PQNU00000000
Rat (Athymic nude, skin)	16–2004	New York, USA (2016)	MSK	PQNQ00000000

a Four *C*. *bovis* isolates displayed a small (~1 mm) in addition to the large (~2 mm) wild-type colony phenotype after subculturing. Colonial size variants were maintained separately and independently sequenced.

^b^NSG; NOD.Cg-Prkdc^scid^ Il2rg^tm1Wjl^/SzJ is a highly immunodeficient mouse model.

### Sequencing, assembly, and annotation of genomes

Genomic DNA was extracted according to the *Quick*-DNA Fungal/Bacterial Microprep Kit (Zymo Research, Irvine, CA, US) protocol, the library was created using the NEBNext Ultra™ DNA Kit (New England BioLabs, Ipswich, MA), and quantified using the Qubit 1.0 (ThermoFisher, Waltham, MA, US). Paired-end sequencing (2×250 bp) was performed with an Illumina MiSeq (Illumina, San Diego, CA, US). PhiX was removed from the FastQ read files with BBDUK version 37.02 using a 31-mer search allowing for a single nucleotide difference in the query, and Trimmomatic version 0.36 was used to remove adapter sequences and discard low quality nucleotides [20]. Cleaned sister reads along with cleaned broken (singleton) reads were provided to SPAdes version 3.11.1 for *de novo* assembly using the ‘—only-assembler’ option [21]. To refine the genome, BWA MEM version 0.7.16a-r1181 was used to map only the cleaned paired reads back onto the assembly with the ‘-x intractg’ option [22], and SAMtools version 1.3.1 generated a binary alignment map (BAM) file [23]. The BAM and assembly files were provided to Pilon version 1.22 and ‘—fix snps,indels—mindepth 0.5’ options were invoked to correct initial assembly errors such as nucleotide polymorphisms, insertions, and deletions which decreased the number of disrupted start and stop codons as well as frameshift mutations [24]. Two subsequent rounds of polishing were performed using the same parameters to correct errors that were missed due to stringent read mapping parameters and conservative correction.

All raw sequence data and assembled genomes have been archived in NCBI’s SRA and GenBank databases (Table 2). NCBI annotated the genomes with their Prokaryotic Genome Annotation Pipeline [25].

**Table 2:** Genomic features of 21 *C*. *bovis* isolates.

Isolate Identifier	Completeness [%]	Coverage [x]	Contigs [#]	Largest contig [bp]	N50	Cumulative length [Mbp]	GC [%]	tRNAs [#]	CDSs [#]	Pseudogenes [#]
**DSM 20582^T^**	98.1	42.0	491	51,655	9708	2.52	72.6	50	2,256	363
**4826**	99.0	86.5	12	1,006,373	522,674	2.67	72.8	53	2,178	109
**4828**	98.6	64.5	16	799,434	274,591	2.63	72.9	52	2,138	103
**MI 82–1021**	97.5	157.1	397	56,959	9,339	2.43	72.4	53	2,167	109
**F6900**	99.1	121.2	31	299,088	152,006	2.63	73.0	52	2,137	122
**WCM1**	98.1	143.6	285	79,948	14,750	2.50	72.7	51	2,132	102
**WCM3-LARGE**	98.6	107.0	331	76,091	12,929	2.51	72.5	52	2,172	96
**WCM3-SMALL**	97.7	136.8	404	75,397	9,881	2.47	72.3	52	2,192	100
**WCM4**	99.0	109.7	245	76,803	16,066	2.59	72.6	53	2,185	94
**WCM5**	97.4	95.2	256	49,900	16,959	2.67	72.8	53	2,318	99
**CUAMC1-LARGE**	95.0	216.6	530	71,161	6,156	2.33	71.8	45	2,161	106
**CUAMC1-SMALL**	95.6	117.2	475	56,803	6,655	2.28	71.8	44	2,078	113
**7894**	93.1	111.5	539	73,531	5,224	2.21	71.6	42	2,031	106
**16-1683-LARGE**	96.9	197.0	398	73,531	9,488	2.45	72.1	48	2,203	127
**16-1683-SMALL**	97.5	119.1	425	73,531	8,900	2.45	72.1	49	2,180	109
**17-0240-LARGE**	98.3	105.3	300	79,813	14,102	2.50	72.3	50	2,193	131
**17-0240-SMALL**	99.0	342.9	133	138,792	34,264	2.66	72.6	51	2,201	128
**16–3465**	99.0	72.4	49	249,836	97,777	2.67	72.6	51	2,179	115
**13–1426**	99.0	86.9	43	297,693	104,613	2.67	72.6	51	2,186	108
**12–5346**	99.0	101.7	38	271,014	138,277	2.67	72.6	51	2,182	106
**16–2004**	99.0	52.3	81	242,233	66,706	2.66	72.6	51	2,185	118

### Species placement within the genus

The Ribosomal Database Project (RDP) was accessed on Aug 2, 2017 to fetch all 16S rRNA genes within the *Corynebacterium* genus. Muscle ver 3.8.1551 was used for alignment, and the ape package (ver 4.1) in R ver 3.3.2 was used to generate a neighbor-joining (NJ) phylogenetic tree with the JC69 substitution model and 1000 bootstraps.

### Genome-wide comparisons and clades within *C*. *bovis*

The average nucleotide identity (ANI) for all 21 *C*. *bovis* genomes, including the Type strain, was computed with BLASTn ver 2.6.0+ and the '-dust no' option using 1 kbp fragments with 200 bp sliding window steps (5x coverage), and only alignment results with at least 30% nucleotide identity and 70% alignment lengths were evaluated. Of those hits, only fragment pairs that still matched each other when the reference and query were swapped were used to calculate the bi-directional average nucleotide identity for each sample pair. K-means clustering was performed with scikit-learn ver 0.19.1 and local maxima were identified with numpy ver 1.13.1. When k was increased from 4 to 6, the two largest groups were both split roughly in halves, so for enhanced resolution, 6 clusters were illustrated in the hierarchical cluster based on Euclidean distances in R ver 3.3.2 with the dendextend ver 1.1.2 and gplots 3.0.1 packages. A second ANI was computed using only the core genes of the 21 *C*. *bovis* isolates using EDGAR version 2.0 (Efficient Database framework for comparative Genome Analyses using BLAST score Ratios), a multiple strain genome comparison software that performs homology analyses based on a specific cutoff that is automatically adjusted to the query dataset [26], based on a BLASTn comparison of the genome sequences described previously [27].

A phylogenetic tree was constructed with EDGAR version 2.0 [26] from concatenated core genes, which has enhanced phylogenetic signal compared to phylogenies derived from single genes such as 16S rRNA genes [28]. Zdobnov and Bork, (2007) recommended the use of all core genes to reinforce the phylogenetic tree [29]. Each set of orthologous genes was individually aligned with MUSCLE [26] and non-matching parts of the alignment were masked by GBLOCKS prior to concatenation of all core genes. The Neighbor-Joining method was chosen for its computational efficiency and interrogative application both necessary for large core genome dataset.

### Map of the circular genomes of *C*. *bovis*

The CGView Comparison Tool (CCT) software package was used for visual comparison of all *C*. *bovis* sequences analyzed [30]. CCT maps consist of rings showing a reference genome and its features and the results of BLAST comparisons of DNA sequences using BLASTn searches and CDS feature translations using BLASTp between the reference and the comparison sequences. A separate BLAST ring was drawn for each comparison genome. A colored arc was drawn beneath the region of the reference sequence to show similarity in the respective comparison ring. The results are presented in the form of graphical maps that can show sequence features, gene and protein names, COG (Cluster of Orthologous Groups of proteins) category assignments, and sequence composition characteristics. Since there is no complete genome for *C*. *bovis*, its phylogenetically closest *Corynebacterium* species, *C*. *falsenii*, (see below) was used as the reference strain as it had a complete sequenced genome.

### The pan- and core genome and singletons of *C*. *bovis* isolates

Directed, subgroup analyses were performed for each of the following datasets: A) All isolates using *C*. *bovis* DSM 20582^T^ as the reference; B) *C*. *bovis* isolates obtained from humans and cattle (which appear to be more closely related to each other than rodents, see Results); and, C) *C*. *bovis* isolates obtained from rodents (mice and a rat). To calculate the pan-genome, core genome, and singletons of the *C*. *bovis* isolates, we used EDGAR version 2.0 [26]. Initially, the annotated sequences were retrieved from GenBank and submitted to EDGAR to create a private project.

The core genome was calculated as the subset of genes presenting orthologs in all the selected isolates and the pan-genome was calculated by adding the entire genome of the first isolate analyzed and subsequent non-orthologous genes from the rest of the isolates [19]. EDGAR was used to determine decay functions predicting the development of the pan-genome with increasing genome number using a nonlinear least squares model fit [26]. Finally, the singletons were calculated as genes that were present in only one strain and did not have orthologs in the other *C*. *bovis* sequenced isolate [19]. Genes are regarded as singletons, if they have no match with a Score Ratio Value (SRV) higher or equal to the master cutoff in any of the isolates in the dataset.

### Core genes classified by cluster of orthologous groups (COGs) functional categories

The COG protein database was generated by classifying genes according to their homologous relationships [31]. Initially, the proteins encoded in seven complete genomes from five phylogenetic lineages were compared for sequence similarities to determine COGs. The COG database has greatly increased temporally as new genomes become available [32]. These COGs are composed of individual orthologous proteins or orthologous sets of paralogs across at least three phylogenetic lineages and represent similar functions [31, 33]. Each COG has a specific functional description (S1 Table), but may also be associated with more than one letter category [33].

The core genome of the combined human and bovine *C*. *bovis* isolates and the core genome of the rodent *C*. *bovis* isolates were determined using EDGAR 2.0 [26]. The singletons for each isolate were also obtained from EDGAR 2.0. Subsequently, the COGs functional categories for each group were determined using the eggNOG (evolutionary genealogy of genes: Non-supervised Orthologous Groups)-mapper [34, 35]. The eggNOG-mapper is a tool for fast functional annotation of new sequences. Functional information is transferred from the eggNOG database using precomputed fine-grained orthologs and phylogenies allowing for a higher precision that excludes paralogs (genes related by duplication within a genome that may have different functions).

### Identification of genomic islands (GEIs), virulence factors, prophages, and CRISPR-Cas systems

Genomic islands (GIs) are gene clusters of likely horizontal origin in bacterial genomes that act as an important stimulant of evolution and can increase the ecological fitness of bacteria by contributing adaptive traits [36]. Furthermore, GIs can carry mobile virulence factors and antimicrobial resistance genes as well as novel genes that confer environmental adaptations.

IslandViewer 4 enables identification and visualization of genomic islands [36]. IslandViewer 4 integrates four different genomic island prediction methods: A) IslandPick based on a comparative genomic approach; B) IslandPath-DIMOB based on nucleotide bias and presence of mobility genes; C) SIGI-HMM based on codon use bias with a Hidden Markov Model approach; and, D) Islander based on the frequent use of tRNA and tmRNA genes as integration sites. Contigs were ordered against a closed reference genome of the closest related species (*C*. *falsenii* DSM 44353^T^, complete genome) using the Mauve contig orderer [37] prior to analysis of all 21 *C*. *bovis* isolates.

VirulentPred was used to determine if *C*. *bovis* isolates contained virulence factors. VirulentPred uses a method based on a bi-layer cascade machine learning classifier (Support Vector Machine, SVM) that includes Position Specific Iterated BLAST (PSI-BLAST) queries [38]. The cascade SVM classifier was shown to be 81.8% accurate in differentiating virulent from non-virulent proteins [38]. For the *C*. *bovis* analysis, we selected a conservative SVM score ≥1.0 to minimize false positive hits while capturing most true positive hits.

The identification of prophages in the genomes was performed using both PHAST (Phage Search Tool) and PHASTER (PHage Search Tool Enhanced Release), which are used to accurately identify, annotate, and graphically display prophage sequences within bacterial genomes or plasmids [39, 40]. Both PHAST and PHASTER use a scoring method to classify prophage regions as intact (> 90), questionable (70–90), or incomplete (< 70). For annotated genomes, PHAST showed 85.4% sensitivity and 94.2% positive predictive value (PPV) and PHASTER showed 86.9% sensitivity and 91% PPV when compared to other prophage identification software [39, 40]. The slight decrease in PPV observed in PHASTER was attributed to parameter adjustments made to increase its sensitivity, which marginally increased the number of false positive results [39]. Given these differences between the two phage search tools, we used both of them.

The identification of CRISPR structures and Cas genes was conducted using CRISPRCasFinder, which allows for accurate definition of direct repeat (DR) consensus boundaries, extraction of the related spacers, and Cas genes [41, 42]. This program has improved specificity compared to its previous version (CRISPRFinder), indicates CRISPR orientation, and uses MacSyFinder to identify Cas genes and the CRISPR-Cas type and subtype. We used the default parameters to find possible CRISPR localizations: a repeat length of 23 to 55 bp, a gap size between repeats of 25 to 60 bp, and a 20% nucleotide mismatch between repeats. We also used the default filters to validate CRISPRs: spacer size from 0.6 to 2.5 the repeat size and the spacers will not be identical (spacer similarity set to 60%) to eliminate tandem repeats.

### Statistical analysis

Each genomic comparative analysis tool utilized includes its own internal algorithm and statistical parameters that have been published and evaluated [26, 27, 30, 34–36, 38, 39, 40, 42]. Where indicated, data were further evaluated by one-tailed unpaired t-tests and expressed as mean ± standard error of the mean (SEM). Values of P < 0.05 were considered statistically significant.

## Results and discussion

### Major genomic features

The genus *Corynebacterium* is highly diverse but some characteristics are common between the different species including having a single, circular chromosome and generally a high GC content [43]. The size of the genomes within *Corynebacterium* range from 1.84 Mbp (*Corynebacterium caspium* DSM 44850^T^, ARBM00000000.1) to 4.7 Mbp (*Corynebacterium variabile* strain NRRL B-4201, GCF_000720035.1). Based on a genome survey of records in PATRIC, many species within the *Corynebacterium* genus, including *C*. *bovis* DSM 20582^T^, lack plasmids, and one such exception is in *Corynebacterium crudilactis* where two distinct plasmid replicons occur [43, 44]. An earlier study evaluated all available *Corynebacterium* species’ genomes and reported they have an average of 2,481 CDSs, with *C*. *caspium* DSM 44850^T^ as the strain with the fewest genes (1,647) and *Corynebacterium aurimucosum* strain 118_CAUR with the most (9,489) [43].

The main genomic features of the 20 *C*. *bovis* isolates sequenced in this study and the Type strain, which was previously sequenced (draft genome), are provided in Table 2. These characteristics are very similar to the other *Corynebacterium* species sequenced [18]. According to QUAST version 4.6.0 and CheckM version 1.0.8, the quality of the genomes is comparable to the only other assembly (*C*. *bovis* DSM 20582^T^, 98.1% completeness) available for the species with a range of 93.1–99.1% (97.8 ± 0.36%) estimated completeness from 585 single-copy gene markers in the *Corynebacterium* genus (n = 80 genomes) [45–47]. The cumulative length of the genome was comparable between isolates including the species Type strain with an average of 2.53 ± 0.03 Mbp, as well as the characteristic high G+C content (72.44 ± 0.09%) reported for this species. The number of tRNAs (50.2 ± 0.7) and the number of CDSs (2,174 ± 12.4) was similar among all isolates [48]. The number of pseudogenes was similar among sequenced isolates (110 pseudogenes ± 2.4), but showed the most difference when compared with the Type strain (363 pseudogenes). The 3.3-fold fewer pseudogenes we observed in our genomes was likely a combination of the Illumina chemistry we used having a lower InDel rate than 454 chemistry (which was used for the Type strain) and our error corrections post-assembly that were not mentioned after the Type strain was assembled [8].

No striking differences in the major genomic features were observed when comparing the sequences of the small and large colony types of the four isolates (one human and three mouse) examined. The average cumulative length of the genomes of the small and large colony types was 2.47 ± 0.08 Mbp and 2.45 ± 0.04 Mbp, respectively. Further, the number of CDSs for the small and large colony types was 2,163 ± 29 CDSs and 2,182 ± 10 CDSs, respectively. A singleton analysis comparing only the four isolates that showed the small and large colony phenotypes showed that the majority of unique genes in each group (i.e., small vs. large type isolates) were classified as hypothetical proteins (14 ± 5.4 small type hypothetical proteins and 8 ± 2.3 large type hypothetical proteins). Although none of the unique genes within members of the large wild-type group showed orthologs, members of the small phenotype group showed two unique orthologous genes: sigma-70 family RNA polymerase sigma factor and DNA polymerase III subunit epsilon, however these genes were only found in two of the four small phenotype members.

These 2 colonial phenotypes were detected in nearly every *C*. *bovis* isolate examined in an earlier study after subculturing [1]. These authors found that the small colony variant phenotype was unstable as subculturing this colony phenotype consistently yielded a mixed population of both small and large colony types. A similar phenomenon occurred with our isolates as well as subculturing of each phenotype that did not consistently yield the same phenotype. Both colony types produced colorimetric biochemical profiles that differed in their enzymatic reactions and carbohydrate utilization [1]. In total, they reported nine different biochemical profiles from isolates confirmed to be *C*. *bovis* using 16S rRNA gene sequencing. Profiles generated most consistently by the small and large colony-types were 4101004 and 0501104, respectively (API Coryne, bioMerieux, Marcy l’Etoile, France). The clinical significance of variations in colony morphology have not been elucidated since these authors found that almost all isolates displayed both phenotypes, whether collected from clinically or subclinically-affected mice.

Small colony variants have been described in several bacterial species and studied extensively among staphylococci [49] where these small colony variants are a slow-growing subpopulation of bacteria with atypical colony morphology, unusual biochemical characteristics, increased antibiotic resistance, and an unstable colonial phenotype [1]. These are all characteristics consistent with the *C*. *bovis* small colony phenotype described in previous work [1]. Small colony variants in other bacterial species have been shown to be identical to the larger colony variant based on 16S rRNA gene sequencing [50]. Furthermore, small colony variants have been associated with persistent, recurrent infections [49] and this phenomenon may support the persistence of *C*. *bovis* observed in mice [1].

### Species placement within the genus

The genus *Corynebacterium*, which had 128 validated species as of February 7^th^, 2018, is highly diversified. It includes pathogenic species that are of medical, veterinary, or biotechnological relevance such as *Corynebacterium diphtheriae*, *C*. *pseudotuberculosis*, and *Corynebacterium ulcerans* [43]. This genus also includes non-pathogenic species of industrial importance, *C*. *glutamicum*, and opportunistic pathogens such as *C*. *bovis*, which affects humans, cattle, and rodents [1–6, 43]. As of April 2018, the PATRIC database had 720 *Corynebacterium* genomes from 90 different species [51, 52].

**Fig 1:**
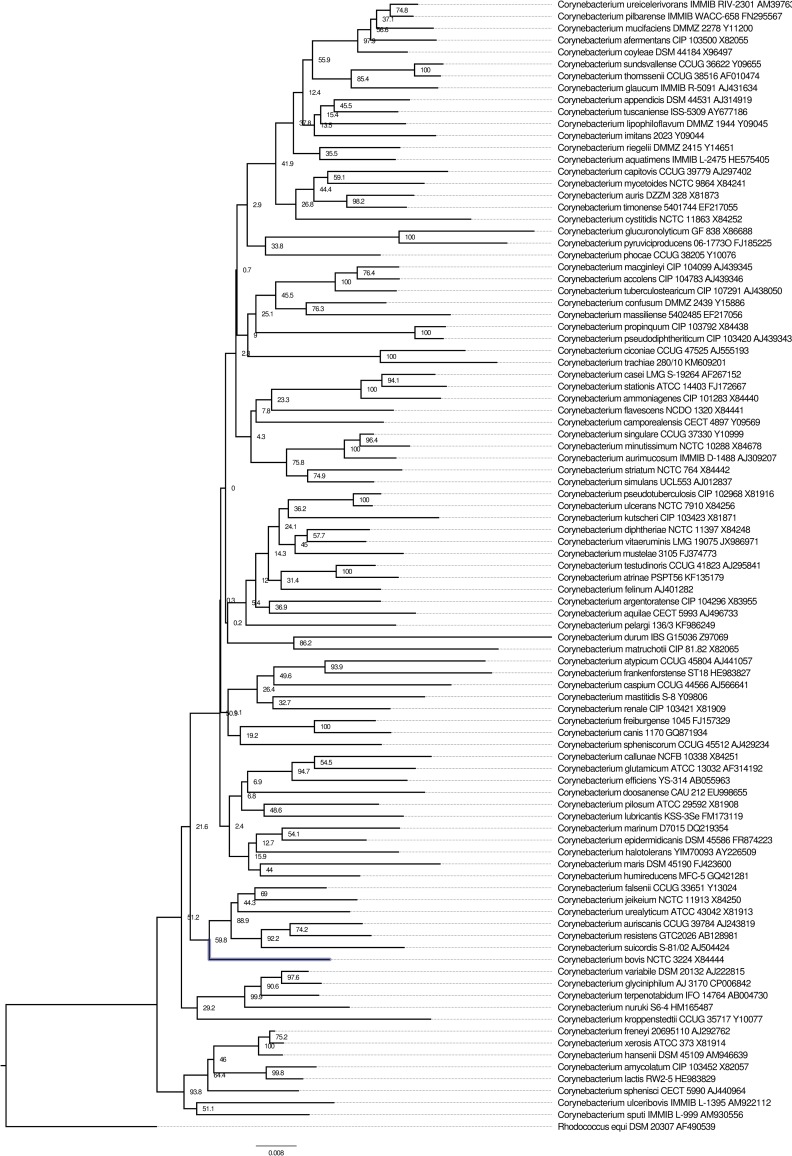
A neighbor-joining phylogenetic tree of 16S rRNA gene sequences from species Type strains within the *Corynebacterium* genus. *Rhodococcus equi* is the rooted outlier. Bootstraps (n = 1,000) are expressed as percentages at each node. The type strain for *C*. *bovis* has its branch highlighted in blue for emphasis. The scale bar represents substitutions per site of the 1,618 total sites including gaps.

We first sought to identify the *Corynebacterium* species most closely related to *C*. *bovis*. All 16S rRNA gene sequences were retrieved for the *Corynebacterium* species Type strains on the RDP site including the *C*. *bovis* Type strain [53]. Of the 128 valid species in the genus, 94 species had near full-length high fidelity sequences, which were used along with the outlier *Rhodococcus equi*. After alignment, a neighbor-joining tree was generated and revealed a clade of six closely related taxa (*Corynebacterium auriscanis*, *Corynebacterium falsenii*, *Corynebacterium jeikeium*, *Corynebacterium resistens*, *Corynebacterium suicordis*, and *Corynebacterium urealyticum*) to *C*. *bovis* (Fig 1). *C*. *falsenii* shared the highest nucleotide similarity (95.9%) of 16S rRNA to *C*. *bovis*. Genome comparisons in some cases showed *C*. *jeikeium* or *C*. *urealyticum* to be a closer neighbor to *C*. *bovis*, however all of these comparisons indicate no species is relatively close to *C*. *bovis* (S1 Fig). With the 16S rRNA gene distance and accessory genome tree showing *C*. *falsenii* being the closest species to *C*. *bovis*, we used it for subsequent analyses as an outlier.

*Corynebacterium falsenii* was first isolated from human blood cultures and cerebrospinal fluid in 1991 and 1995, respectively [47]. The species has also been isolated from an infant with bacteremia where *C*. *falsenii* was isolated from blood cultures from a central intravenous line and central line catheter tip after systemic vancomycin therapy to treat a wound infected with methicillin-resistant *Staphylococcus aureus* (MRSA), *Streptococcus agalactiae*, and *Bacteroides tectus* [54]. However, the clinical significance of *C*. *falsenii* remains largely unknown, as it is rarely recovered from human clinical material. Furthermore, this bacterium has been isolated from the respiratory tracts of eagles and black storks, from bioaerosols sampled in duck houses, and from the cloacal microbial community of black-winged stilts [55–57]. Thus, *C*. *falsenii* may be a member of the natural microflora of wild and domesticated birds. The genome sequence of *C*. *falsenii* DSM 44353^T^ included a circular chromosome of 2,677,607 bp (63.18% G+C content) and a circular corynephage ɸCFAL8171I genome of 42,009 bp (61.74% G+C content) [47]. An identical linear copy of ɸCFAL8171I was present in the chromosome as a prophage, which suggested, based upon the circularized phage genome, that this corynephage had entered a lytic cycle in a subpopulation of the culture used to prepare the genomic DNA for WGS [47]. For the purpose of our study, the completed genome of the type strain of *C*. *falsenii* (DSM 44353^T^) was subsequently used as an outlier and reference for *C*. *bovis* comparative analyses.

### Genome-wide comparisons and groups within *C*. *bovis*

**Fig 2:**
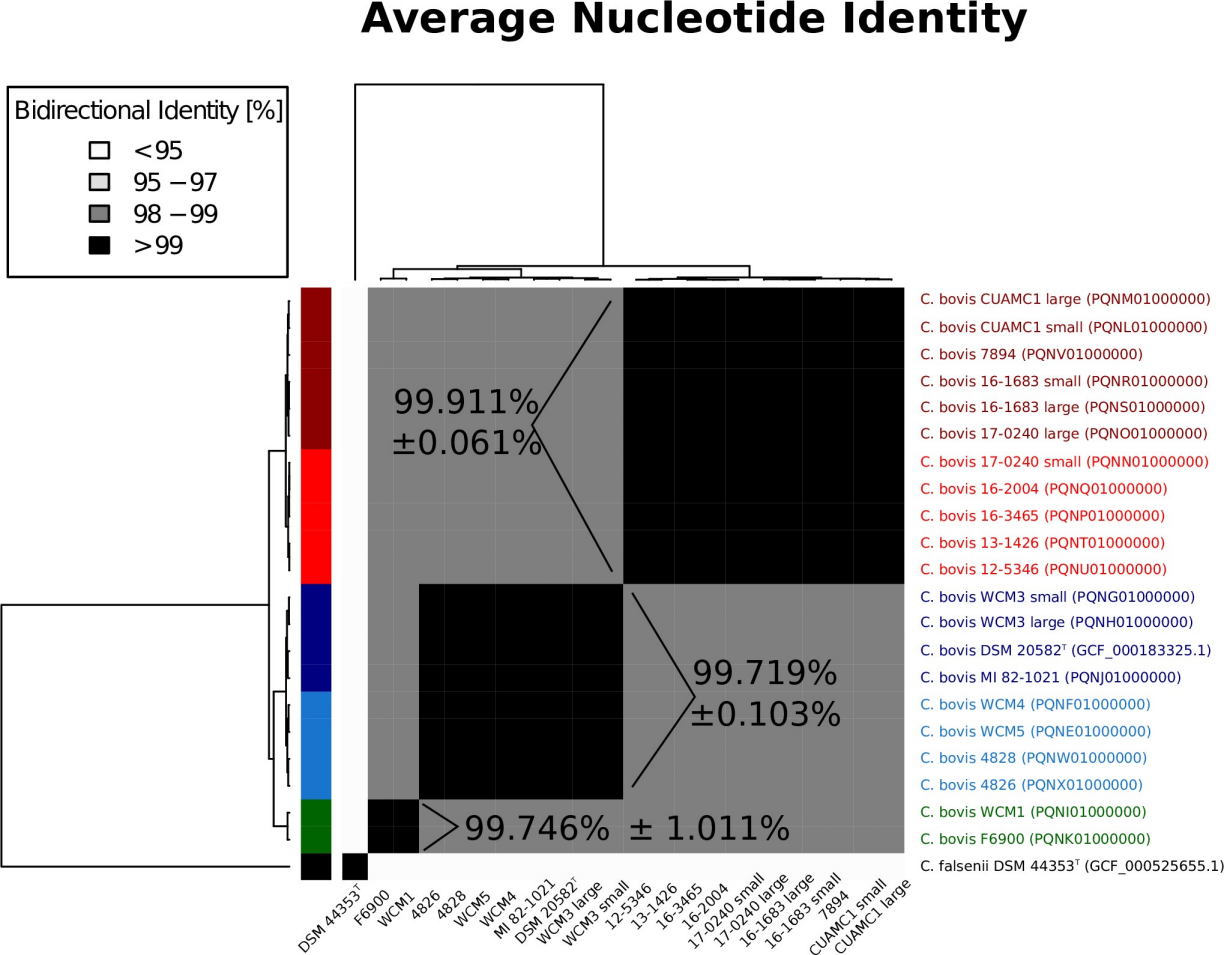
Genomes clustered according to pairwise average nucleotide identity (ANI) distances. Each pairwise ANI result is represented in a greyscale square, with none occurring between 95–97%. *Corynebacterium falsenii* is shown as a neighboring outlier to the *C*. *bovis* genomes (n = 21). Six clusters are color-coded in the left dendrogram (black, green, blue, dark blue, red, and dark red). When the genomes are clustered into just four groups, the blue and dark blue clusters collapse into one group, and the red and dark red clusters merge as well. ANI percentages shown for three select groups represent arithmetic means and standard deviations of bi-directional pairs.

ANI was computed for each genome pair, and the bi-directional (or "orthologous") values were used for each of the 231 comparisons. An ANI of ≤95% is often used as a cutoff for species demarcation [58], and in all cases we observed <95% ANI in pairs containing the *C*. *falsenii* isolate (76.043% minimum; 76.615% maximum). Such distant ANI values to its nearest neighbor and such similar values within *C*. *bovis* (98.854% ± 0.826%) provide strong support for classifying *C*. *bovis* as a genomically distinct *Corynebacterium* species. Unsupervised clustering based on these identity values indicated the optimal number of genome groups was 2 with a silhouette score of 0.915. This was expected, because the clustering placed *C*. *falsenii* separately from all *C*. *bovis* isolates. Therefore, we used the next best quantity of genome groups, which was when the number of clusters (k = 4) gave a 0.837 silhouette score. Interestingly, when 4 clusters are formed from the genomes, isolates were clustered according to the pathogen's host. Isolates clustered in red in Fig 2 are all from rodents (mice and a rat), while the blue and more closely related green clusters are all from humans and cows. Each of these groups have highly similar ANI values (>99.7%) with tight, low deviations within each group, and different identity values (approximately 98%) between groups. The high percent identity observed within isolates from the same host suggest there may be differentiable host-specific loci and if a new *C*. *bovis* isolate were to be sequenced, one could infer its origin. Whether such nucleotide differences were a large array of short sequences scattered throughout the chromosome or were made up of only a few but large loci requires additional analyses.

**Fig 3:**
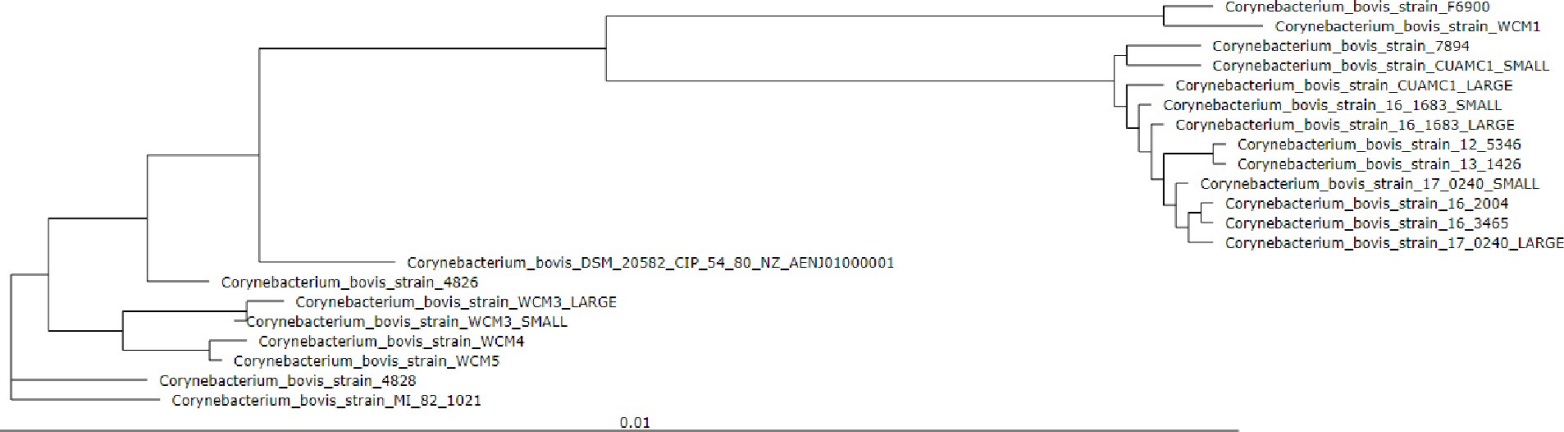
Phylogenetic tree for 21 *C*. *bovis* genomes. The tree was built of a core of 1,354 genes per genome, 28,434 in total. The core has 598,172 amino acid-residues/bp per genome, 12,519,612 in total. Tree re-rooted.

A phylogenetic tree for the 21 *C*. *bovis* genomes was built of a core of 1,354 genes per genome (Fig 3). This tree showed a similar clustering to the ANI calculations where the rodent isolates were classified closer to each other. The two human isolates (F6900 and WCM1) formed a distinct subgroup and the rest of the human isolates were grouped with the bovine isolates. To confirm this clustering observation, an ANI heatmap matrix was computed based on a BLASTn comparison of the genome sequences using EDGAR version 2.0 (S2 Fig). This showed a similar pattern to the first ANI confirming that although all isolates had a high bidirectional percent identity (ANI >95%), which corroborates they are all the same species, there are small differences that appeared to be host-associated. These potential differences were further explored in subsequent analyses.

### Map of the circular genomes of *C*. *bovis*

**Fig 4:**
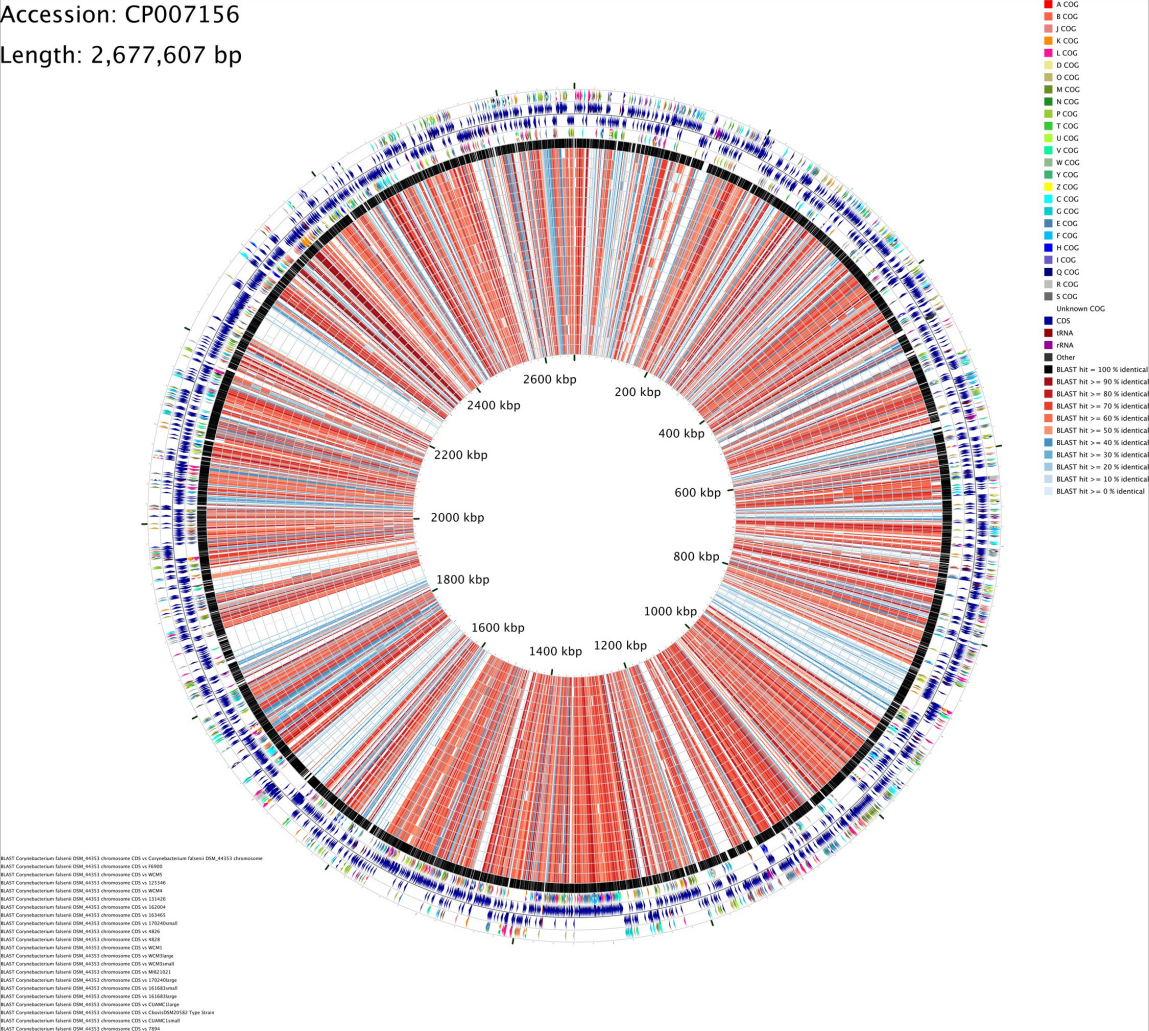
Comparative genomic map of 21 *C*. *bovis* isolates using the CGView Comparison Tool (CCT). All isolates were aligned using *C*. *falsenii* DSM 44353^T^’s complete genome as a reference. From the inner to the outer circle: *C*. *bovis* 7894, CUAMC1-Small, DSM 20582^T^, CUAMC1-Large, 16-1683-Large, 16-1683-Small, 17-0240-Large, MI 82–1021, WCM3-Small, WCM3-Large, WCM1, 4828, 4826, 17-0240-Small, 16–3465, 16–2004, 13–1426, WCM4, 12–5346, WCM5, F6900, *C*. *falsenii* DSM 44353^T^ (black circle). These rings represent regions of sequence similarity detected by BLAST comparisons of DNA sequences using BLASTn searches and CDS feature translations using BLASTp conducted between the reference genome and the 21 *C*. *bovis* comparison genomes. Colored arrows represent COG functional categories. Blue arrows represent sequence features. Direction of the arrows represent either the forward or the reverse strand. CDS, coding sequences; tRNA, transfer RNA; rRNA, ribosomal RNA.

A circular genome comparison of the 21 *C*. *bovis* isolates was performed with CCT software using *C*. *falsenii* DSM 44353^T^’s genome as the reference (Fig 4). The rings represent regions of sequence similarity detected by BLAST comparisons conducted between CDS translations from the reference and the 21 *C*. *bovis* genomes compared. The genomes are plotted from outer to inner circles by order of decreasing similarity to the reference. The reference genome is included as one of the comparison genomes as it served to reveal portions of the reference that are unable to produce BLAST hits due to ambiguous bases, BLAST filtering, or an absence of protein-coding sequences [30]. The most labile or divergent portions of a reference genome stood out as light-colored regions adjacent to the reference ring, while well-conserved portions of the reference showed dark-colored arcs that form spikes of conservation extending towards the center of the map. This methodology also revealed genome segments whose similarity is inconsistent with the general trends presented in the map.

### The *C*. *bovis* pan-genome

**Fig 5:**
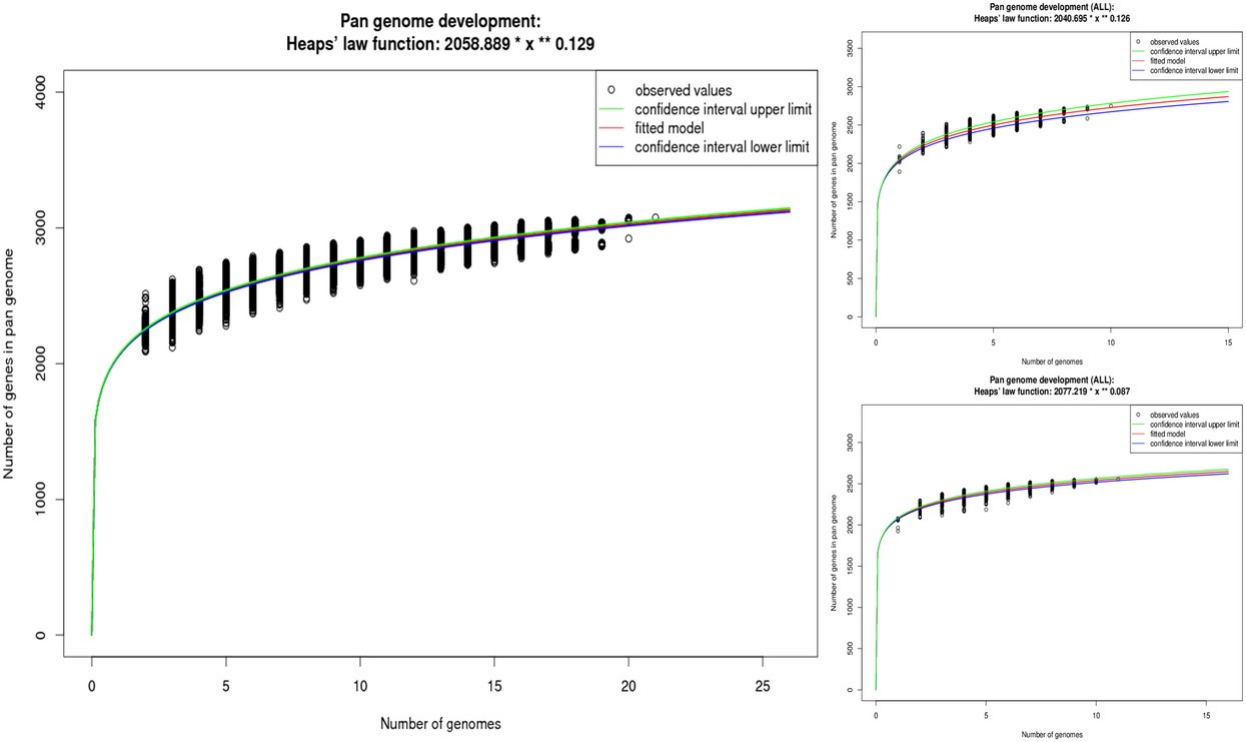
Pan-genome development of *C*. *bovis*. Pan-genome development using permutations of all 21 isolates of *C*. *bovis* (left); the pan-genome development of the *C*. *bovis* isolates obtained from humans and cows (upper right); and, the pan-genome development of the *C*. *bovis* isolates obtained from rodents (upper right). The red curve shows the fitted exponential Heap’s law function, blue and green curves indicate the upper and lower boundary of the 95% confidence interval.

We calculated the pan-genome, i.e., the total number of non-redundant genes, to obtain a general assessment of the gene repertoire in *C*. *bovis* using the software EDGAR version 2.0 (Fig 5) [19]. The pan-genome of *C*. *bovis* contained a total of 3,067 genes, which is 1.47-fold greater than the average total number of genes in each of the 21 isolates (2,091). However, when the pan-genome of the isolates obtained from human and bovine hosts were calculated separately from the isolates obtained from rodents, a mild difference appeared, in which the isolates from humans and cows had a pan-genome of 2,747 genes, 1.32-fold greater than the average total number of genes in each human/bovine isolate (2,082), and the isolates from rodents had a pan-genome with 2,556 genes, 1.24-fold greater than the average total number of genes in each rodent isolate (2,056).

Comparative genomic analyses have showed considerable intra-species variability among genomes within a species for many bacterial species [59]. Given this observation, determining how many genomes need to be sequenced to capture a species entire gene-repertoire becomes crucial. One way to obtain this information is to establish the number of new genes each time a new genome is sequenced and added to the analysis [59]. This can be estimated using the Heaps’ law function, which is an empirical law used to describe the number of distinct genes (*n*) that grow according to a sub-linear power law of the number of genomes considered (*N*). That is, *n* ~ *N*^ɣ^, with 0 < ɣ < 1. In other words, the rate at which new genes are found decreases as more genomes are added to the analysis, as this rate is proportional to N^(ɣ-1)^ = N^-α^, with α = 1 - ɣ. Thus, addition of new genes becomes increasingly difficult as sampling continues.

The extrapolation of the *C*. *bovis* pan-genomes was calculated by curve fitting based on Heaps’ law, as represented by the formula *n* = *k* * *N*^-α^, where *n* is the expected number of genes for a given number of genomes, *N* is the number of genomes, and the other terms are constants defined to fit the specific curve [19, 59]. The variables *k* and ɣ were determined to be 2,058.89 and 0.129, respectively, using EDGAR version 2.0. According to Heaps’ Law: A) an α ≤1 is representative of an open pan-genome which means that new genes will keep being added as more genomes are analyzed and the pan-genome will increase, and B) an α >1 represents a closed pan-genome where it will not be significantly affected with the addition of new genomes. Following previously established methodology [19] and using the formula α = 1 - ɣ, we determined that the pan-genome of *C*. *bovis* is increasing with an α of 0.87, indicating that it has an open pan-genome similar to their study with *C*. *pseudotuberculosis*. The pan-genome was also separately estimated for isolates obtained from humans and cows and from rodents. The isolates from humans and cows had the same α as the entire pan-genome (0.87); however, the isolates from rodents had a higher α of 0.91. Although these values are approaching a constant as more genomes are sampled, i.e., approaching an α >1 and considered a closed pan-genome, additional genomes appear to be needed to capture the entire gene repertoire for *C*. *bovis*. It is important to note that all isolates used in this study were obtained from hosts showing clinical signs, thus non-pathogenic isolates were excluded from this analysis and may, in part, explain the open pan-genome. Thus, non-pathogenic isolates, such as the “non-hyperkeratosis-associated coryneform” obtained from asymptomatic nude mice in previous work [15], could contribute to the species genomic diversity.

### The *C*. *bovis* core genome

**Fig 6:**
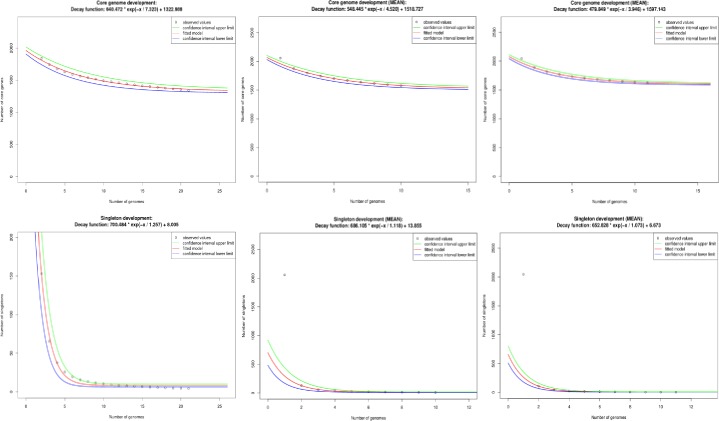
*C*. *bovis’* core genome and singleton development. The core genome development using permutations of all 21 isolates of *C*. *bovis* (upper left); the core genome development of the *C*. *bovis* isolates obtained from humans and cattle (upper-center); the core genome development of the *C*. *bovis* isolates obtained from rodents (mice and a rat) (upper-right); the singleton using permutations of all 21 isolates of *C*. *bovis* (lower left); the singleton development of the *C*. *bovis* isolates obtained from humans and cattle (lower-center); and, the singleton development of the *C*. *bovis* isolates obtained from rodents (mice and a rat) upper-right).

**Fig 7:**
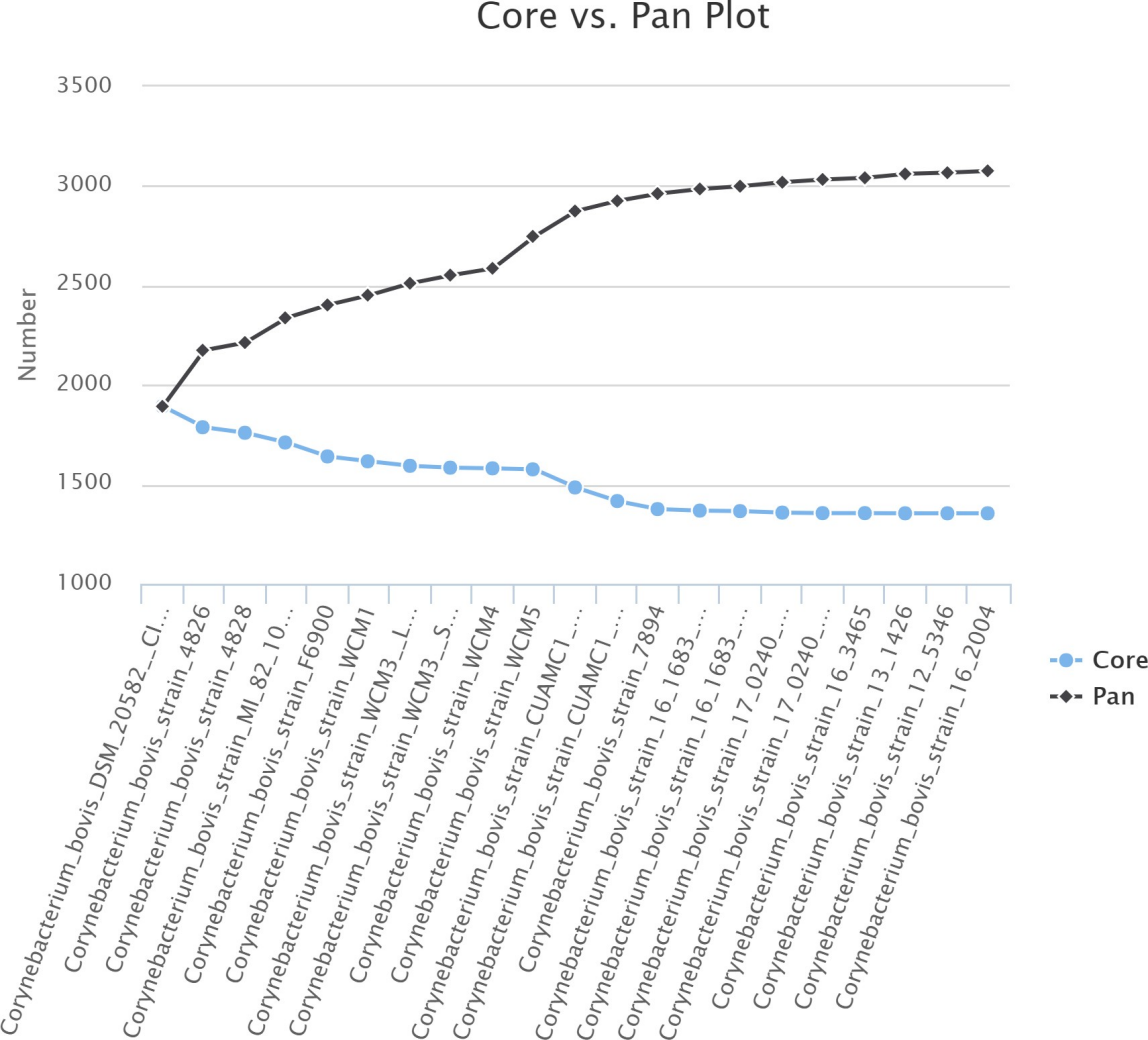
Plot of the pan-genome vs. core-genome for each of the 21 *C*. *bovis* isolates. The number of core genes is reflected in blue and the pan-genome in black for each of the isolates.

A species’ core genome is defined as the subgroup of genes from the pan-genome that are shared by all strains [19]. Thus, the core genome encodes proteins necessary for basic biological and phenotypic functions associated with the species. We confirmed relatively few pseudogenes existed for the assemblies we contributed to GenBank (Table 1) which was important because the artificial disruption of coding sequences would otherwise yield an unexpectedly smaller core genome. Similar to previous studies, *C*. *bovis’* core genome was calculated using Edgar version 2.0 by defining the subgroup of genes that presented orthologs in all the isolates using the SRV method [19]. There were 1,354 core genes, which represented 44% of the pan-genome (3,067 genes) and may decrease slightly by the inclusion of new genomes as shown in Figs 6 and 7. The authors explained that “the extrapolation of the curve can be calculated by the least-squares fit of the exponential regression decay to the mean values, as represented by the formula *n* = *k* * *exp[-x/τ]+tg(ϴ)*, where *n* is the expected subset of genes for a given number of genomes, *x* is the number of genomes, *exp* is Euler’s number, and the other terms are constants defined to fit the specific curve” [19]. This method predicts that with a large number of genomes (*x*), the k * exp[-x/τ] term will tend toward 0, where *tg(ϴ)* represents the merging of the genome subgroup. Based on this observation, *C*. *bovis’* core genome converged at 1,323 genes, which represented 43% of the species’ pan-genome.

**Fig 8:**
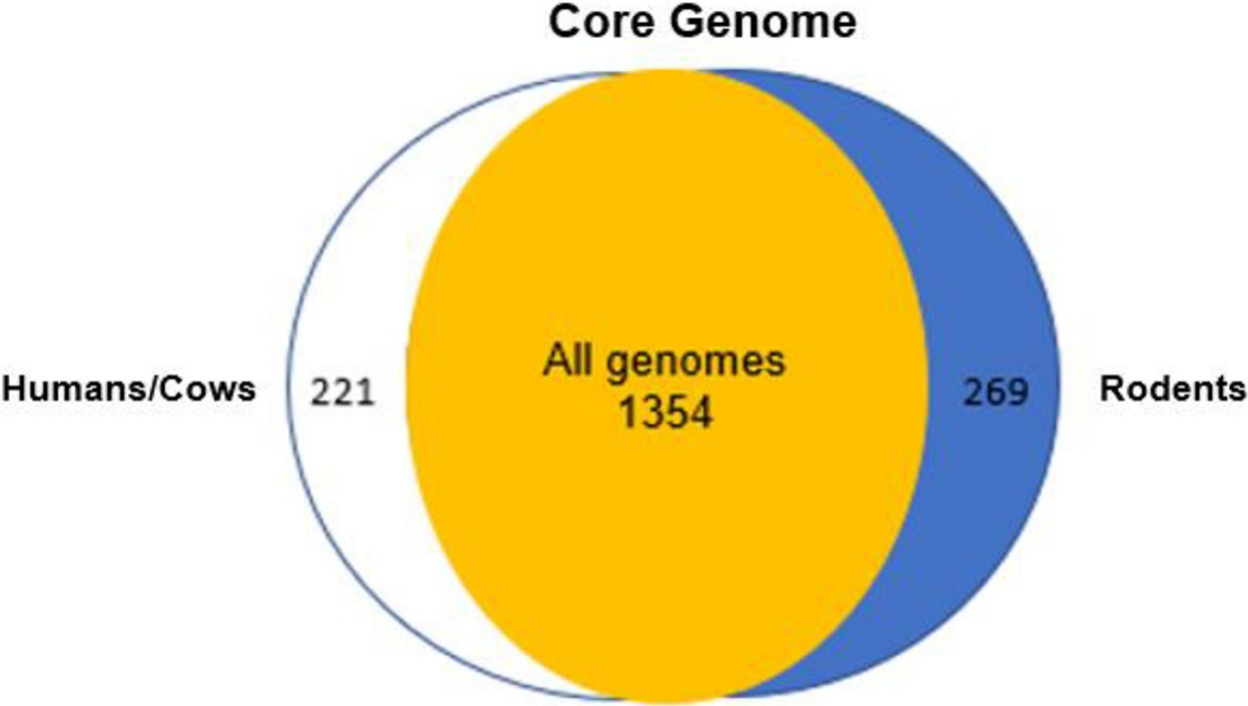
Venn diagram depicting the number of core genes in segregated groups of *C*. *bovis* isolates. All genomes, the number of genes composing the core genome of all 21 isolates; Humans/Cows, the number of genes of the core genome of the *C*. *bovis* isolates obtained from humans and cows, which were absent in one or more of the *C*. *bovis* isolates obtained from rodents; Rodents, the number of genes of the core genome of the *C*. *bovis* isolates obtained from mice and a rat, which were absent in one or more of the *C*. *bovis* isolates obtained from humans or cows.

Analysis of the core genomes of human and cow isolates as compared to those from rodents is presented in Fig 6. This analysis revealed that the core-genome from humans and cows was 1,575 genes, and stabilized at approximately 1,519 genes when evaluated by exponential regression decay. The isolates from rodents had a less compact core genome of 1,623 genes, which stabilized at 1,597 genes. The human/cow isolates are predicted to contain 221 orthologous genes that are shared by strains from this group of isolates and are absent from one or more of the rodent isolates (Fig 8). The rodent isolates, with 1,623 genes, contained 269 core genes that were absent from one or more of the human/cow isolates (Fig 8).

**Fig 9:**
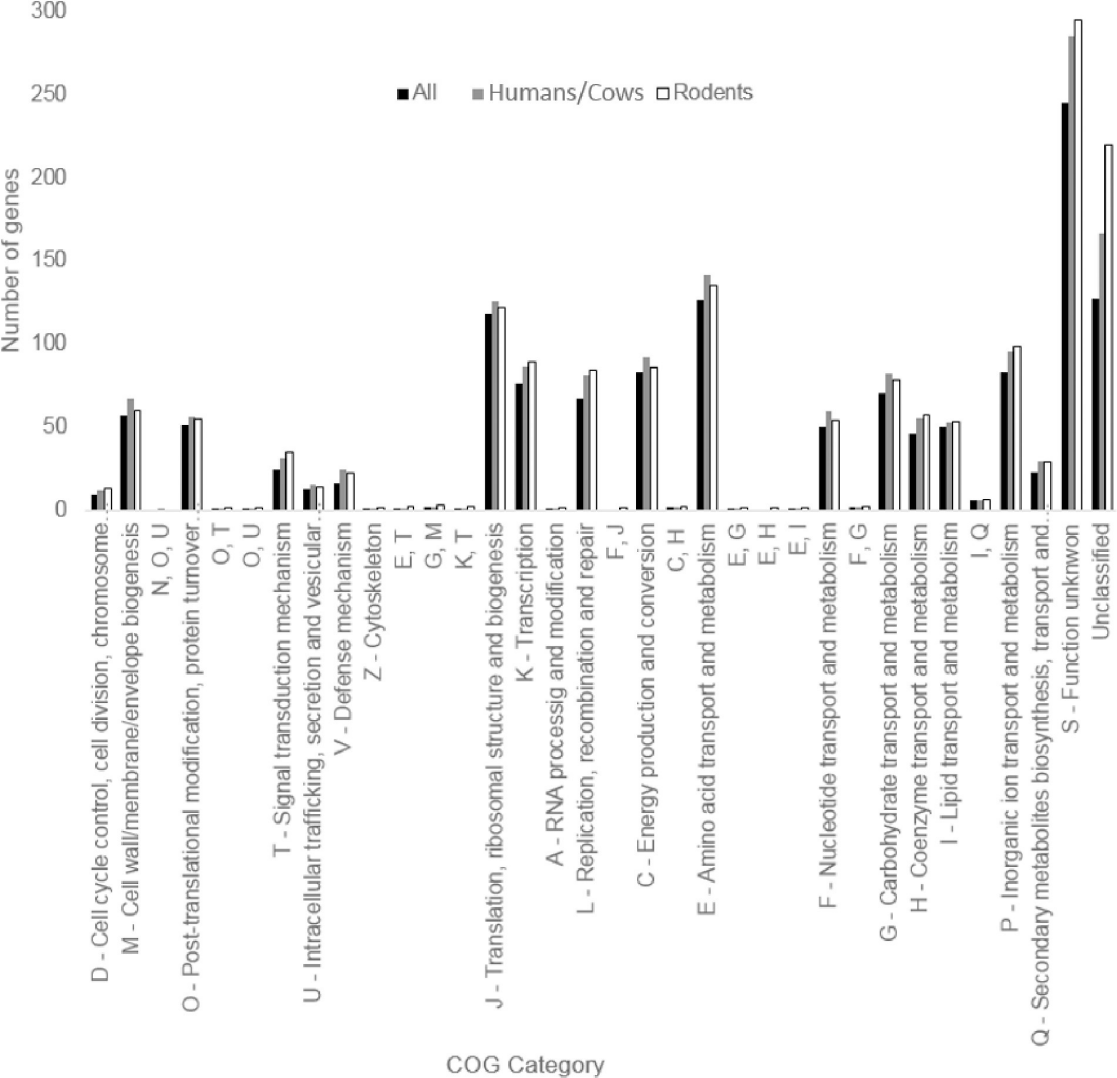
Number of genes classified in various COG categories for *C*. *bovis* isolates obtained from humans and cows vs. isolates obtained from rodents.

The core genome of all the isolates and the differential core genome of the human/cow and rodent isolates were classified by COG functional category. As reflected in Fig 9, the core genome of all the isolates had a large number of genes in the categories ‘‘Metabolism” (e.g., energy production and conversion, amino acid, nucleotide, carbohydrate, and lipid transport and metabolism) and ‘‘Information storage and processing” (e.g., RNA processing and modification, chromatin dynamics, translation, transcription, replication, recombination, and repair). This is comparable to a similar analysis conducted for *C*. *pseudotuberculosis* strains [19]. A large proportion of the core genome of all the isolates was classified as ‘‘Function unknown” or “Unclassified”. However, when analyzing the differential core genes of the human/cow and rodent isolates separately, a higher proportion of ‘‘Function unknown” or “Unclassified” genes was detected in the differential core genes when compared with the core genome of all the isolates (Fig 9).

*C*. *bovis* rodent isolates had a pan-genome of 2,556 genes and a core genome of 1,623 genes or almost two thirds (63.5%) of those in the pan-genome. In contrast, the *C*. *bovis* isolates from humans/cows pan-genome contained 2,747 genes, with a core genome of 1,575 genes or slightly higher than half (57.3%) of the genes in the pan-genome. The latter reflects the high genetic variability found in this group of isolates. In comparison, previous work on a different *Corynebacterium* sp. found a very low percentage (42.5%) of core genomes in a group of *C*. *pseudotuberculosis* biovar *equi* isolates, which is one of the lowest reported for any bacterial species [18]. As a point of reference, *Escherichia coli’s* core genome is 44%, *Pseudomonas syringae* is 64%, *Streptococcus pneumonia* is 74%, and *Listeria monocytogenes* is 80% of their respective pan-genomes [18].

### Singletons: Isolate-specific genes detected in *C*. *bovis*

An isolate’s singletons are unique genes absent from all other isolates and thus increase the number of genes in the pan-genome [19]. We used the SRV method and EDGAR to determine the subgroup of *C*. *bovis* singletons as the genes that did not present orthologs in any other isolate (Table 3).

**Table 3:** Number of genomic islands, putative virulence factors, and CRISPR-Cas systems in 21 *C*. *bovis* isolates.

Isolate ID	Host	Genomic islands [#]	Putative virulence factors [#]	CRISPR-Cas system [#]	CRISPR-Cas type-subtype
**DSM 20582**^**T**^	Bovine	11	7	1	Type I-E
**4826**	Bovine	8	33	1	Type I-E
**4828**	Bovine	4	6	1	Type I-E
**MI 82–1021**	Bovine	5	19	1	Type I-E
**F6900**	Human	2	19	1	Type I-E
**WCM1**	Human	1	17	1	Type I-E
**WCM3-LARGE**	Human	9	28	0	N/A
**WCM3-SMALL**	Human	4	21	0	N/A
**WCM4**	Human	9	39	2	Type I-E
**WCM5**	Human	6	22	2	Type I-E
**CUAMC1-LARGE**	Rodent	18	44	1	Type I-E
**CUAMC1-SMALL**	Rodent	13	54	1	Type I-E
**7894**	Rodent	7	29	1	Type I-E
**16-1683-LARGE**	Rodent	16	25	1	Type I-E
**16-1683-SMALL**	Rodent	13	33	1	Type I-E
**17-0240-LARGE**	Rodent	12	28	2	Type I-E
**17-0240-SMALL**	Rodent	17	63	2	Type I-E
**16–2004**	Rodent	16	75	2	Type I-E
**16–3465**	Rodent	15	56	1	Type I-E
**13–1426**	Rodent	12	76	1	Type I-E
**12–5346**	Rodent	12	61	2	Type I-E

We used the least-squares fit of the exponential regression decay to the mean values, *n* = *k* * *exp[-x/τ]+tg(ϴ)*, to calculate *tg(ϴ)* for three datasets: A) All 21 genomes; B) human/bovine isolates; and, C) the rodent isolates (Fig 6). The *tg(ϴ)* for all the genomes was 8.005, indicating that each sequenced genome added approximately eight genes to *C*. *bovis’* total gene pool. Individual analysis of the two groups of isolates revealed that each sequenced human/bovine isolate contributed ~14 genes but each sequenced rodent isolate contributed approximately seven genes.

The average number of singletons in human/cow isolates (6.6 ± 0.85) was significantly higher (n = 10, unpaired t-test one-tailed, *P* < 0.01) than the average number of singletons in rodent isolates (1.9 ± 0.39) (S2 Table). Thus, the singletons are contributing, at least in part, to the genomic variability of the former. The singletons were classified into categories of the COGs using the eggNOG-mapper. Similar to *C*. *pseudotuberculosis* isolates, most *C*. *bovis’* singletons were not classified into COGs (S3 Fig) and therefore their biological functions are unknown [18]. One of the rodent isolates had a singleton involved with transcription. Regardless of their biological function these singletons contribute to the species’ diversity and likely confer selective advantages such as niche adaptation, antibiotic resistance, and the ability to colonize new hosts.

### Prophage presence in *C*. *bovis* isolates

No intact (completeness score <90) or questionable (completeness score 70–90) prophages were found in any of the 21 *C*. *bovis* isolates using either PHAST or PHASTER. Using PHAST, at least one incomplete (completeness score <70) phage region was identified in all isolates (range 1–5 phage regions/isolate). The average completeness score was low (27.6 ± 1.6) thus most likely do not represent complete prophage candidates. Interestingly, PHASTER, which has mildly higher sensitivity, only identified one incomplete prophage region in *C*. *bovis* WCM3 in both the large and small colony phenotype genomes. The completeness score of this prophage region was 10 (out of 150). The length of the region was 7.2 kbp. The total number of proteins was 10 (six matching the phage protein database, three matching the bacterial database, and one hypothetical protein without a match in the databases) and the start and end positions within the genome were 70486–77687 (S3 Table). The phage with the highest number of proteins most similar to those identified in this region was *Mycobacterium* phage ArcherNM (NC_031277; Siphoviridae; dsDNA virus; genome length: 5.3 kbp; number of proteins: 91) with four similar proteins. The percentage of GC nucleotides of the region was 59.48%. The proteins were either identified as hypothetical proteins or phage-like proteins. No lysis, protease, coat protein, tail shaft, attachment site, integrase, or transposase proteins characteristic of phages were identified in this region.

These results are surprising given the abundance and ubiquity of phages in bacterial populations [60]. There are several known antiphage systems found in bacteria acquired through evolution or lateral transfer [61]. These include surface alterations to block phage adsorption, inhibition of phage DNA penetration, DNA restriction/modification (RM) systems, acquiring phage-specific immunity through CRISPRs and abortive infection (Abi) [60–64]. Some of these antiphage systems (see below) may be extremely effective and could play critical roles in keeping *C*. *bovis* free of phages.

### Detection of genomic islands, pathogenicity factors, and CRISPR-Cas systems

**Fig 10:**
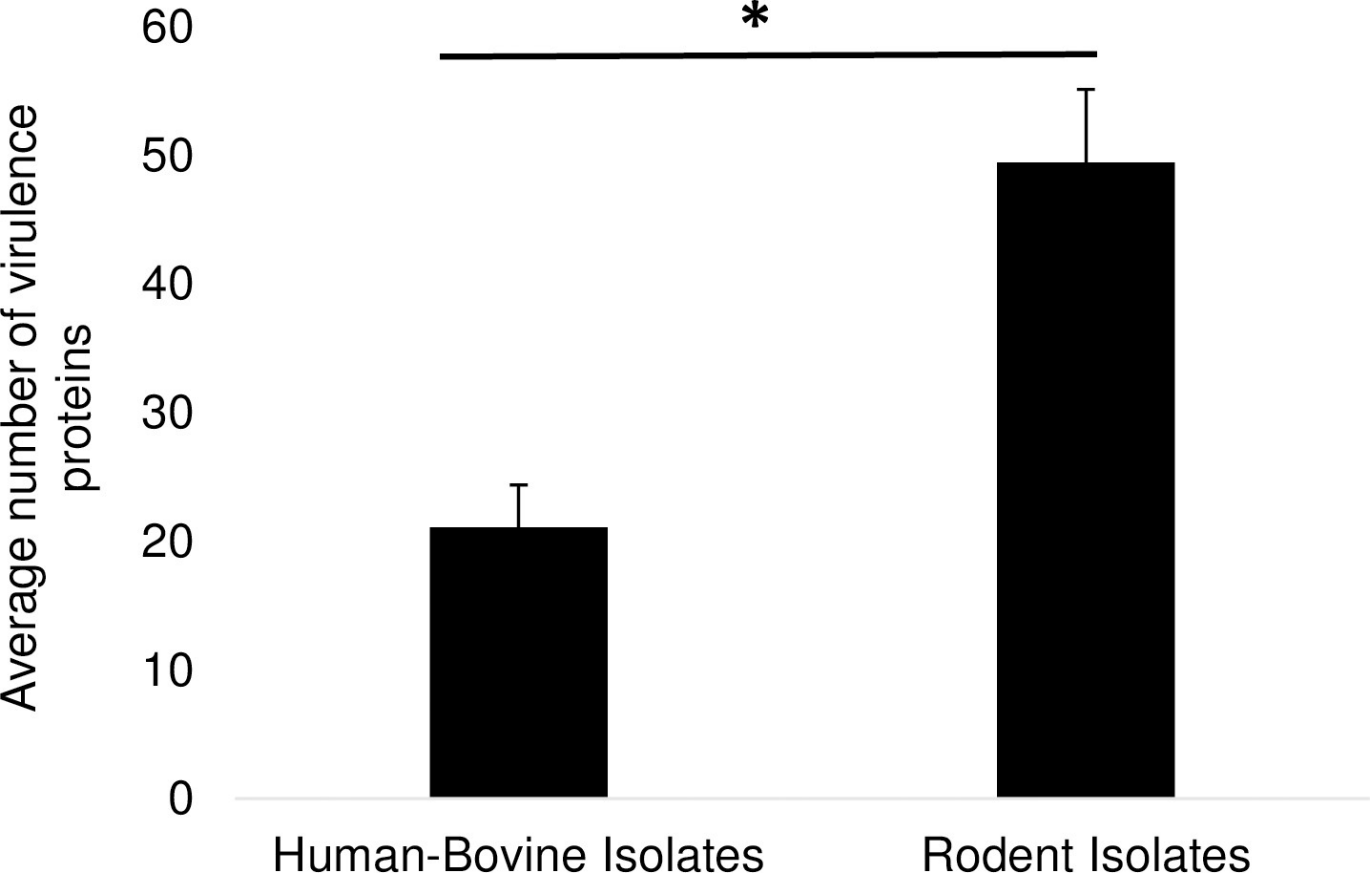
Virulence proteins in *C*. *bovis* based on the host from which it was isolated: Humans and cows (n = 10) and rodents (n = 11). *p <0.001, unpaired t-test one-tailed. Error bars represent standard error of the mean.

The average number of genomic islands was significantly higher (n = 10, unpaired t-test one-tailed, *P* <0.001) in the rodent isolates (13.7 ± 0.94) compared to the human/bovine isolates (5.9 ± 1.04) (Table 3). The average number of putative virulence genes was significantly higher (n = 10, unpaired t-test one-tailed, *P* <0.001) in the rodent isolates compared (49.5 ± 5.65) to the human/bovine isolates (21.1 ± 3.27) (S4 and S5 Tables; Fig 10).

Genomic islands may arise from horizontal gene transfers which results in intraspecies genome plasticity, facilitating an accelerated evolutionary process [19]. Pathogenicity islands (PAIs) are a class of GIs that carry virulence genes, i.e., “factors that enable or enhance the growth of an organism inside a host” [19]. Virulence factors work in conjunction to promote optimal colonization, production of offspring, and propagation of bacteria [65]. Although some virulence factors with more general roles are also found in nonpathogenic bacteria (e.g., hydrolases, transporters, and chelators), most, such as toxins and secretion systems, are highly specialized and influenced by the adaptation of the pathogen to its host niche.

Closer examination of the virulence factors identified by the VirulentPred software revealed several toxins associated with *C*. *bovis*. The following toxins were found in three or more of the human/cow isolates: type II toxin-antitoxin (TA) system HicA family toxin; addiction module antidote protein, HigA family; TA system subunit antitoxin, Txe/YoeB family addiction module toxin; and, type II TA system mRNA interferase toxin, RelE/StbE family; and peptidyl-prolyl isomerase (Table 4). All of these virulence factors, except toxin HicA, were only found in human/cow isolates. With respect to the rodent isolates, type II toxin-antitoxin system HicA family toxin; salicylate synthase (only found in rodent isolates); uncharacterized toxins; and, transcriptional regulators were found in three or more isolates.

**Table 4:** Virulence factors found in 3 or more *C*. *bovis* isolates, their function, and associated microorganisms where these factors have been found.

Virulent factor	Function	Associated example organisms	Reference
*Type II TA system: HicA family toxin*	Induces cleavage of mRNA and tmRNA (transfer-messenger RNA) thereby preventing translation; bacteriostatic; generation of persister cells (bacterial persistence); RNA degradation	*Escherichia coli*; *Burkholderia pseudomallei*	[68, 79]
*Type II TA system: RelE/StbE family*	mRNA interferase; induction of SOS response; associated with multiple toxin families (including HigA)	*Streptococcus pneumoniae*; *Escherichia coli; Proteobacteria*	[66]
*Addiction module: HigA family antitoxin*	Counteract growth inhibition (affect translation); associated with multiple toxin families (including RelE)	*Escherichia coli; Proteobacteria; Firmicutes*	[66]
*TA system subunit antitoxin, Txe/YoeB family addiction module toxin*	Endonuclease activity; nucleic acid phosphodiester bond hydrolysis; RNA catabolic process	*Sutterella wadsworthensis;* *Neisseria* bacilliformis; *Desulfobacter curvatus*; *Anaerolineae* bacterium; *Actinomyces* sp.; *Desulfovibrio* sp.; *Acaryochloris* sp.	[80]
*Peptidyl-prolyl isomerase*	Enzyme catalyzing the rate-limiting protein folding step at peptidyl bonds preceding proline residues within polypeptide chains; calcineurin sequestering (immune system function); gene expression, signal transduction, protein secretion, development, tissue regeneration, and virulence-associated protein (macrophage infectivity potentiator-like)	*Escherichia coli; Legionella pneumophila; Yersinia pseudotuberculosis; Salmonella enterica* serovar *Enteritidis*; *Shigella flexneri*; *Helicobacter pylori*; Ubiquitous in prokaryotes and eukaryotes	[65, 74]
*Salicylate synthase*	Biosynthesis of salicylate (building block of siderophores [organic ferric-chelators] and nonsteroidal anti-inflammatory)	*Mycobacterium tuberculosis; Yersinia enterocolitica*; *Y*. *pestis; Pseudomonas aeruginosa*; *E*. *coli; Serratia marcescens*	[75–77]
*Transcriptional regulators*	Regulation of virulence factor expression	*Streptococcus pyogenes*	[73]

*Corynebacterium bovis* isolates have various TA systems. TA systems are widely distributed in eu- and archae-bacteria and are composed of small genetic modules found on mobile genetic elements and bacterial chromosomes and tend to be associated with plasmid maintenance [63, 66]. TA systems are divided into 3 classes based on the nature of the antitoxin and its mode of action: antitoxins of type I and III systems are small RNAs that inhibit either toxin expression (type I) or activity (type III), and antitoxins of type II systems, such as those detected in several *C*. *bovis* isolates, are proteins that inactivate toxins by forming protein–protein complexes [66]. The number of type II systems have shown high inter- and intra-species variability. Plasmids have evolved mechanisms to avoid plasmid-free cells that act by killing plasmid-free daughter cells using a strategy known as post-segregational killing or addiction and is executed by Type I and II systems. Because antitoxins are less stable than toxins, bacteria that do not inherit a plasmid copy, shift the balance between toxins-antitoxins and the less labile toxin becomes free from inhibition leading to cell death. Thus, the bacterial cell is described as addicted to antitoxin production and TA genes and participate in plasmid stabilization. TA systems also play an antiphage role as mediators of phage abortive infection (Abi) mechanisms [62, 67]. Abis are activated by phages and interfere with metabolic processes that inhibit cellular function [60]. They can function in any step between phage DNA penetration and cell lysis. Some of these mechanisms can interfere with phage DNA replication, phage RNA transcription, interact with phage genes, reduce synthesis of phage structural proteins, or cause premature bacterial cell death. Abi systems lead to death of the infected cell as an altruistic gesture to protect the surrounding clonal population from predation [60, 61, 64, 67]. The toxins target central cellular processes such as translation, replication and cytoskeletal/cell wall formation by inhibiting DNA gyrase and causing mRNA degradation. It is hypothesized that free toxin, which has a longer half-life than its antitoxin, is released from TA systems during the degradation of host DNA or the shutdown of host transcription which results in cell death and disruption of phage multiplication [60]. Our work and others [43] have not identified any plasmids associated with *C*. *bovis*, thus it appears that the principal function of the TA systems found in this species is phage protection.

One interesting TA system found in three human, a bovine, and two rodent *C*. *bovis* isolates was a member of the HicA family toxin. HicA toxins have been associated with the formation of antibiotic tolerant (persister) cells that may play a role in chronic and recurrent disease [68]. Persister cells were first identified in a *Staphylococcus aureus* subpopulation that survived supra-lethal doses of antibiotic demonstrating a biphasic killing pattern [69]. This subpopulation was not a genetically defined group as subsequent growth and exposure to antibiotics yielded a similar frequency of survivors indicating that this tolerance was most likely due to phenotypic variation within the population. The current hypothesis proposes that persister cells, although genetically identical to susceptible bacteria, represent phenotypic variants with differences in gene expression that can also be affected by environmental cues [70]. Persister cells could act as a reservoir for chronic infections and have been demonstrated in a large number of species including *E*. *coli*, *Burkholderia pseudomallei*, *Streptococcus mutans*, *Pseudomonas aeruginosa* and *Mycobacterium tuberculosis* [68, 70–72]. Although the molecular mechanisms that generate persister cells are not fully understood, some evidence shows that TA systems are involved by causing growth arrest (dormancy) and increasing the number of persister cells tolerant to antimicrobials [68]. These toxins can interact with cellular components such as RNA, ribosomes or DNA gyrase, resulting in a bactericidal or bacteriostatic cellular response. The ubiquity of *C*. *bovis* and the inability to eradicate it from animal research facilities housing immunocompromised rodents [1, 2] might, in part, be explained by the phenomenon of persister cells.

Other commonly detected virulence factors included transcriptional regulators, which were found in all rodent and several human/cow *C*. *bovis* isolates. These virulence-related regulators can guide a coordinated response by incorporating external parameters, such as nutrient availability, chemical stressors, host immune components, and temperature, with information on the pathogen’s metabolic state and signals from the expressed genome [73]. Furthermore, these transcriptional regulators can help evade host immunity by responding to nonpathogenic metabolic inputs such as carbohydrate levels. Another virulence factor, peptidyl-prolyl isomerases, which were found in a cow and two human *C*. *bovis* isolates, are enzymes that catalyze the rate-limiting protein folding step at peptidyl bonds preceding proline residues within polypeptide chains [65, 74]. However, there is evidence of virulence-associated functions within this family of proteins. Principally, they assume secondary virulence roles by facilitating, for example, assembly of outer membrane proteins such as pilus/fimbriae components, siderophore receptors, and adhesins. Finally, salicylate synthase, which was only found in some rodent *C*. *bovis* isolates, is involved in the biosynthesis of salicylate [75–77]. Some bacteria use salicylate as a building block in the biosynthesis of siderophores, organic ferric-chelators, such as yersiniabactin in *Yersinia pestis* and *Y*. *enterocolitica*, pyochelin in *P*. *aeruginosa*, mycobactin in *M*. *tuberculosis* and enterobactin in *E*. *coli* [76, 78]. To support their metabolism, many pathogenic bacteria and fungi commonly use siderophores to obtain iron, an essential mineral for growth, from the host [75, 76]. Siderophores are exposed to the surrounding environment where they bind to iron molecules and then re-enter the bacterial cell [76]. Given the prevalence of siderophore systems in pathogenic microorganisms, therapies that inhibit its biosynthesis could prove useful.

CRISPR-Cas systems were identified in all but two (WCM3 small and large colony) *C*. *bovis* isolates (Table 3). All systems showed a high accuracy score (evidence level = 4; highest level possible) based on parameters used by the CRISPRCasFinder program that assigns an evidence level rating (1–4) that evaluates repeat and spacer similarity [41]. Furthermore all isolates, including WCM3 small and large, contained several CRISPR systems with lower evidence levels that most likely represent small CRISPR-like structures (i.e., have only two or three direct repeats [DRs]) [42]. Many of these structures are not true CRISPRs and need to be critically investigated. However, the identification of confirmed structures from these low evidence level CRISPRs may help to better understand how new CRISPRs are created, their evolution and dissemination.

Previous findings showing that spacers appear to derive from bacteriophages and proteins from Cas genes have a similar function to eukaryotic RNA interference systems suggests that CRISPR systems serve as prokaryotic adaptive immunity against genetic aggressions [58, 64, 81, 82, 83]. All CRISPR-Cas systems found in *C*. *bovis* were classified as Type I-E. In Type I systems, the precursor CRISPR RNA (pre-crRNA) is processed by CRISPR specific endoribonucleases into small crRNAs which are then bound to Cas proteins to guide the recognition and cleavage of complementary DNA sequences [84, 85]. Type I-E CRISPR-Cas systems are encoded by a single operon that contains *cas1*, *cas2*, and *cas3* together with the genes for the subunits of the Cascade complex [84]. Past work has demonstrated that Type I-E systems uses a base pairing-independent mechanism that recognizes four fixed protospacer adjacent motifs (PAMs) sequences in the target DNA [85]. PAMs are conserved sequences in the invader genome located next to the target sequence but are never found in the host CRISPR loci thus allowing it to discriminate non-self invader DNA from self DNA (i.e., invader sequences) found in the CRISPR locus.The combination of CRISPR-Cas and TA systems in *C*. *bovis* could help explain why no intact prophages were found in any of the isolates analyzed.

## Conclusions

Limited genomic information was available for *C*. *bovis* as only a single draft genome was available on NCBI. Next-generation, high-throughput DNA sequencing techniques, combined with new computational advances in assembly, annotation, and comparative analysis, provide the ability to study a larger number of bacterial species and isolates. These new capabilities provide significant advantages for identifying differences that may exist between bacterial isolates.

In this study, 20 new genomes of the opportunistic pathogen *C*. *bovis* were sequenced and assembled in high-quality scaffolds with an average size of 2.53 Mbp. These genomes revealed molecular characteristics that were very similar to the only other sequenced *C*. *bovis* genome (*C*. *bovis* DSM 20582^T^).

*Corynebacterium bovis* isolates obtained from human and cow hosts showed greater genetic similarity than those obtained from rodents which, based on their molecular characteristics, formed a distinct clade. These results support, in part, our hypothesis that isolates from different hosts would be genomically distinct. Characterization of *C*. *bovis’* pangenome revealed that its genetic variability was greater than previously recognized. The number of genomic islands and virulence factors was significantly higher in the rodent isolates, which carry an extensive and diverse repertoire of virulence factors, e.g., type II toxin-antitoxin systems, peptidyl-prolyl isomerase, and salicylate synthase, which shape the host-pathogen interaction. All isolates had several low level CRISPR systems and all but two isolates had at least one complete CRISPR-Cas system, which may partly explain why no intact prophages were found in any isolate as CRISPRs function in prokaryote adaptive immunity. A large number of the virulence factors identified in *C*. *bovis* were only characterized as “toxins” and the majority of the singletons detected in its’ pangenome were uncharacterized emphasizing the importance of characterizing bacterial proteins with unknown functions.

## Supporting information

S1 Table Cluster of orthologous genes (COGs) functional categories and letter associations. (PDF)

S2 Table Number of singletons in each of the 21 *C. bovis* isolates. (PDF)

S3 Table CDS position and BLAST hit of the genes identified within the incomplete prophage found in both large and small colony isolates of *C. bovis* WCM3 (human isolate). (PDF)

S4 Table All virulence factors identified in 10 *C. bovis* isolates obtained from human and bovine hosts. (PDF)

S5 Table All virulence factors identified in 11 *C. bovis* isolates obtained from rodent hosts. (PDF)

S1 Fig Genome-wide comparisons of *C. bovis* to near neighbors. Neighbor-joining (A) core and (B) accessory genome trees from protein clustering with an 2.0 inflation value using mcl v14-137 in roary v3.12.0 on Type strain protein pairs with at least 40% identity. Bootstrap values that exceed 70% are shown. Scale bars represent nucleotide substitutions per site and the fraction of genes absent per total accessory genes (respectively). (C) Dendrogram from hierarchical clustering of 1,378 pairwise ANI comparisons of all publicly available assemblies for select *C*. *bovis* neighbors. Genomes were clustered (color-coated) the same way as Fig 2. Species names listed are those from NCBI despite some appearing to be incorrectly labeled (e.g., strain 1055 CURE) or novel (e.g., strain 355 CFAL).
(TIF)

S2 Fig ANI heatmap matrix for 21 *C. bovis* isolates. The method is based solely on the core genome’s BLASTn comparisons.
(TIF)

S3 Fig Number of unique genes classified in COG categories of *C. bovis* isolates obtained from humans and cows vs. isolates obtained from rodents. (TIF)
