# Supplementary material for: Genotypic differences between strains of the opportunistic pathogen Corynebacterium bovis isolated from humans, cows, and rodents
Source: PLoS One. 2018 Dec 26;13(12):e0209231. doi: 10.1371/journal.pone.0209231 (PMC6306256; doi:10.1371/journal.pone.0209231)
Supplement: S1 Table — (PDF) [file pone.0209231.s001.pdf]

**S1 Table. Cluster of orthologous genes (COGs) functional categories and letter associations**.

| Cellular processes and signaling | Information storage and processing | Metabolism | Poorly characterized |
|---|---|---|---|
| **[D]** Cell cycle control, cell division, chromosome partitioning | **[A]** RNA processing and modification | **[C]** Energy production and conversion | **[R]** General function prediction only |
| **[M]** Cell wall/membrane/envelope biogenesis | **[B]** Chromatin structure and dynamics | **[E]** Amino acid transport and metabolism | **[S]** Function unknown |
| **[N]** Cell motility | **[J]** Translation, ribosomal structure and biogenesis | **[F]** Nucleotide transport and metabolism | |
| **[O]** Post-translational modification, protein turnover, and chaperones | **[K]** Transcription | **[G]** Carbohydrate transport and metabolism | |
| **[T]** Signal transduction mechanisms | **[L]** Replication, recombination and repair | **[H]** Coenzyme transport and metabolism | |
| **[U]** Intracellular trafficking, secretion, and vesicular transport | | **[I]** Lipid transport and metabolism | |
| **[V]** Defense mechanisms | | **[P]** Inorganic ion transport and metabolism | |
| **[W]** Extracellular structures | | **[Q]** Secondary metabolites biosynthesis, transport, and catabolism | |
| **[Y]** Nuclear structure | | | |
| **[Z]** Cytoskeleton | | | |
