# Supplementary material for: Genotypic differences between strains of the opportunistic pathogen Corynebacterium bovis isolated from humans, cows, and rodents
Source: PLoS One. 2018 Dec 26;13(12):e0209231. doi: 10.1371/journal.pone.0209231 (PMC6306256; doi:10.1371/journal.pone.0209231)
Supplement: S2 Table — (PDF) [file pone.0209231.s002.pdf]

S2 Table. Number of singletons in each of the 21 *C. bovis* isolates.

Human and Cow Isolates	Singletons [#]	Rodent Isolates	Singletons [#]
DSM 20582^T	11	CUAMC1-SMALL	4
MI 82-1021	9	CUAMC1-LARGE	3
F6900	8	16-1683-SMALL	3
WCM1	8	17-0240-SMALL	3
4826	7	16-1683-LARGE	2
WCM3-LARGE	6	17-0240-LARGE	2
WCM4	6	7894	2
WCM5	6	13-1426	1
4828	3	16-3465	1
WCM3-SMALL	2	16-2004	0
		12-5346	0
