# Supplementary material for: Genotypic differences between strains of the opportunistic pathogen Corynebacterium bovis isolated from humans, cows, and rodents
Source: PLoS One. 2018 Dec 26;13(12):e0209231. doi: 10.1371/journal.pone.0209231 (PMC6306256; doi:10.1371/journal.pone.0209231)
Supplement: S3 Table — (PDF) [file pone.0209231.s003.pdf]

S3 Table. CDS position and BLAST hit of the genes identified within the incomplete prophage found in both large and small colony isolates of *C. bovis* WCM3 (human isolate).

BLAST Hit	Database
PHAGE_Mycoba_Xeno_NC_031243: hypothetical protein; CXF37_00300; phage(gi100030)	Virus/Prophage
Hypothetical protein; CXF37_00305	Bacterial
Antitoxin HicB; CXF37_00310	Bacterial
PHAGE_Mycoba_Phrann_NC_031266: hypothetical protein; CXF37_00315; phage(gi100034)	Virus/Prophage
PHAGE_Salmon_vB_SosS_Oslo_NC_018279: error-prone lesion bypass DNA polymerase V; CXF37_00320; phage(gi399528790)	Virus/Prophage
PHAGE_Cronob_ENT39118_NC_019934: protein umuD; CXF37_00325; phage(gi431811072)	Virus/Prophage
PHAGE_Gordon_CaptainKirk2_NC_031072: hypothetical protein; CXF37_00330; phage(gi100036)	Virus/Prophage
peptidylprolyl isomerase; CXF37_00335	Bacterial
MerR family transcriptional regulator; CXF37_00340	Bacterial
PHAGE_Mycoba_ArcherNM_NC_031277: hypothetical protein; CXF37_00345; phage(gi100035)	Virus/Prophage
