# Supplementary material for: Genotypic differences between strains of the opportunistic pathogen Corynebacterium bovis isolated from humans, cows, and rodents
Source: PLoS One. 2018 Dec 26;13(12):e0209231. doi: 10.1371/journal.pone.0209231 (PMC6306256; doi:10.1371/journal.pone.0209231)
Supplement: S4 Table — (PDF) [file pone.0209231.s004.pdf]

S4 Table. All virulence factors identified in 10 *C. bovis* isolates obtained from human and bovine hosts.

Virulent factors	DSM 20582	MI821021	4826	4828	F6900	WCM1	WCM3L	WCM3S	WCM4	WCM5
Hypothetical proteins	7	15	28	4	13	11	19	16	29	15
Toxin-antitoxin system subunit antitoxin				1					2	1
TXE/YOEB family addiction module toxin				1					2	1
MERR family transcriptional regulator										1
Isochorismatase										1
Very short patch repair endonuclease										1
Type II toxin-antitoxin system RELE/PARE family toxin									2	1
Transcriptional regulator									2	1
Addiction module antidote protein, HigA family		1	1			2	2	1	2	
Toxin HicA			1		1		2	1		
Type II toxin-antitoxin system mRNA interferase toxin, RELE/STBE family		1					2	1		
Peptidylprolyl isomerase		1					2	1		
Mbth family protein								1		
Flavin reductase			1				1			
Multidrug ABC transporter ATP-binding protein						1				
DNA-binding protein					1	1				
Xre family transcriptional regulator					1	1				
Atp-binding protein						1				
Transposase		1								
Nucleoid-associated protein Isr2			1							
COPG family transcriptional regulator			1							
Toxin					1					
Ribonuclease P protein component					1					
50s ribosomal protein l34					1					
