# Supplementary material for: Genotypic differences between strains of the opportunistic pathogen Corynebacterium bovis isolated from humans, cows, and rodents
Source: PLoS One. 2018 Dec 26;13(12):e0209231. doi: 10.1371/journal.pone.0209231 (PMC6306256; doi:10.1371/journal.pone.0209231)
Supplement: S5 Table — (PDF) [file pone.0209231.s005.pdf]

S5 Table. All virulence factors identified in 11 *C. bovis* isolates obtained from rodent hosts.

Virulent Proteins	CUAMC 1S	CUAMC 1L	17-0240S	17-0240L	16-3465	16-2004	16-1683S	16-1683L	12-5346	13-1426	7894
Hypothetical protein	44	36	57	25	46	68	29	22	53	64	25
Transcriptional regulator	2	1	2	1	2	3	1	1	1	2	1
Toxin HicA									1	2	
MbtH family protein	1										
DNA-binding protein									1	2	
XRE family transcriptional regulator										2	
Toxin	1	1	2		1	1		1	1	2	
Ribonuclease P protein component											1
salicylate synthase	1	1		1							
UDP-N-acetylenolpyruvoylglucosamine reductase	1										
DUF2252 domain-containing protein	1										
Type II toxin-antitoxin system HicA family toxin	2	2	2		2	3	2	1	2	2	1
TIGR02391 family protein	1						1				
D-alanyl-D-alanine carboxypeptidase/D-alanyl-D-alanine-endopeptidase		1									
Ornithine monooxygenase		1		1							
Serine protease		1									
Molybdenum cofactor biosynthesis protein					1						
Molybdopterin synthase sulfur carrier subunit					1						
Molybdopterin-guanine dinucleotide biosynthesis protein A;					1						
DUF4177 domain-containing protein					1						
WhiB family transcriptional regulator					1						
XRE family transcriptional regulator									1		
Shikimate dehydrogenase									1		
50S ribosomal protein L34											1
