# Supplementary figures and images for: Genotypic differences between strains of the opportunistic pathogen Corynebacterium bovis isolated from humans, cows, and rodents
Source: PLoS One. 2018 Dec 26;13(12):e0209231. doi: 10.1371/journal.pone.0209231 (PMC6306256; doi:10.1371/journal.pone.0209231)

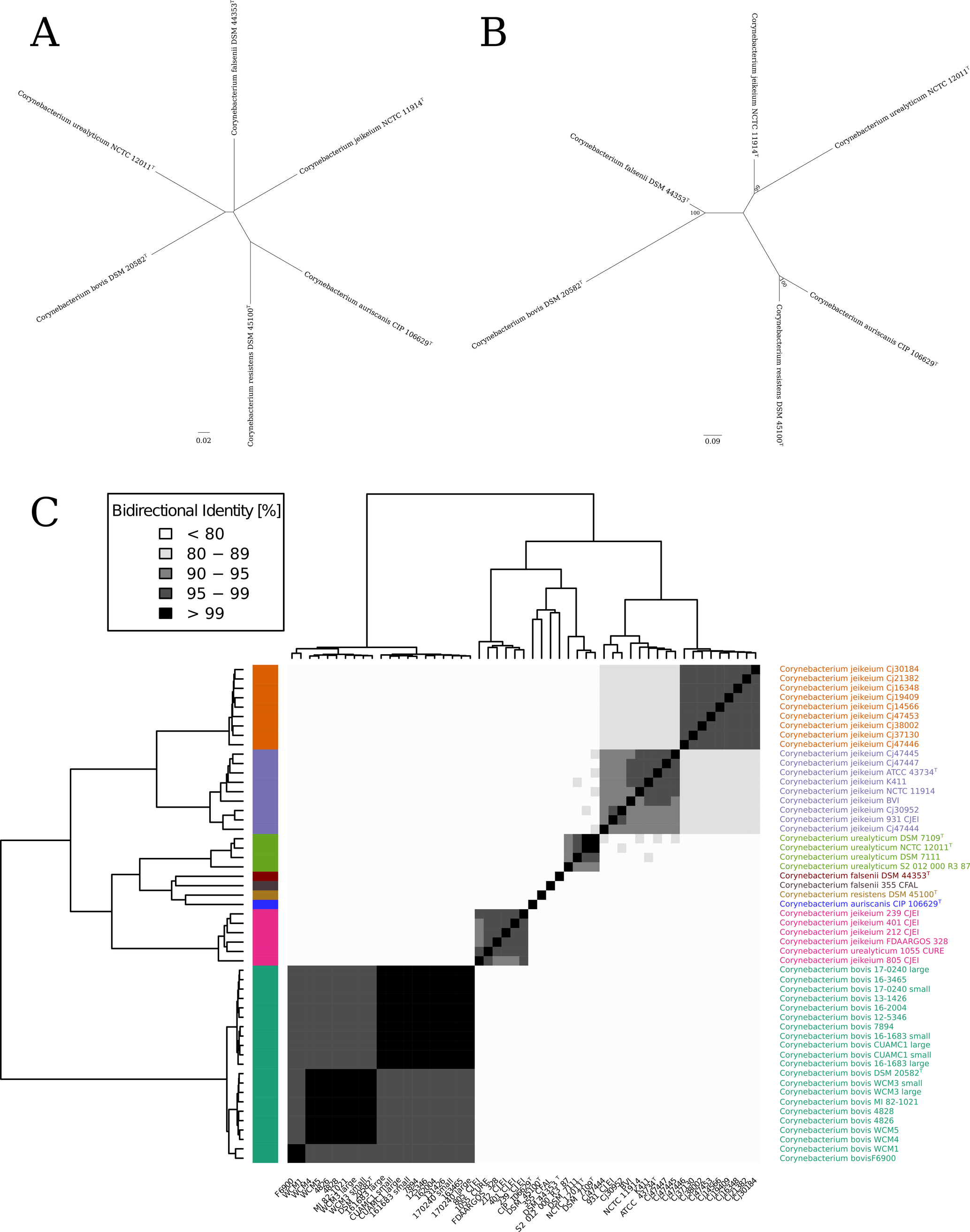

Supplement: S1 Fig — Neighbor-joining (A) core and (B) accessory genome trees from protein clustering with an 2.0 inflation value using mcl v14-137 in roary v3.12.0 on Type strain protein pairs with at least 40% identity. Bootstrap values that exceed 70% are shown. Scale bars represent nucleotide substitutions per site and the fraction of genes absent per total accessory genes (respectively). (C) Dendrogram from hierarchical clustering of 1,378 pairwise ANI comparisons of all publicly available assemblies for select C. bovis neighbors. Genomes were clustered (color-coated) the same way as Fig 2. Species names listed are those from NCBI despite some appearing to be incorrectly labeled (e.g., strain 1055 CURE) or novel (e.g., strain 355 CFAL). (TIF) [file pone.0209231.s006.tif]

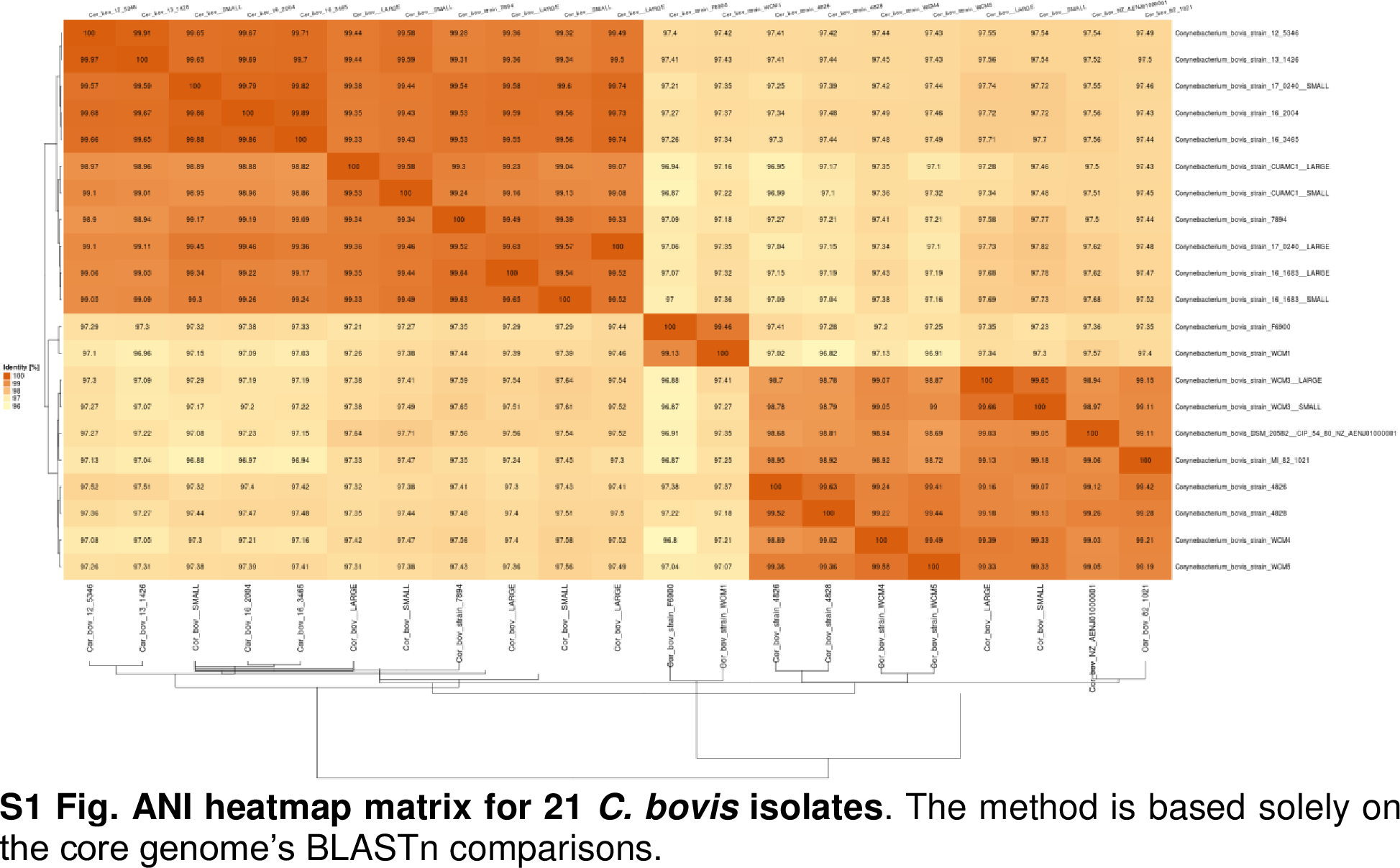

Supplement: S2 Fig — The method is based solely on the core genome’s BLASTn comparisons. (TIF) [file pone.0209231.s007.tif]

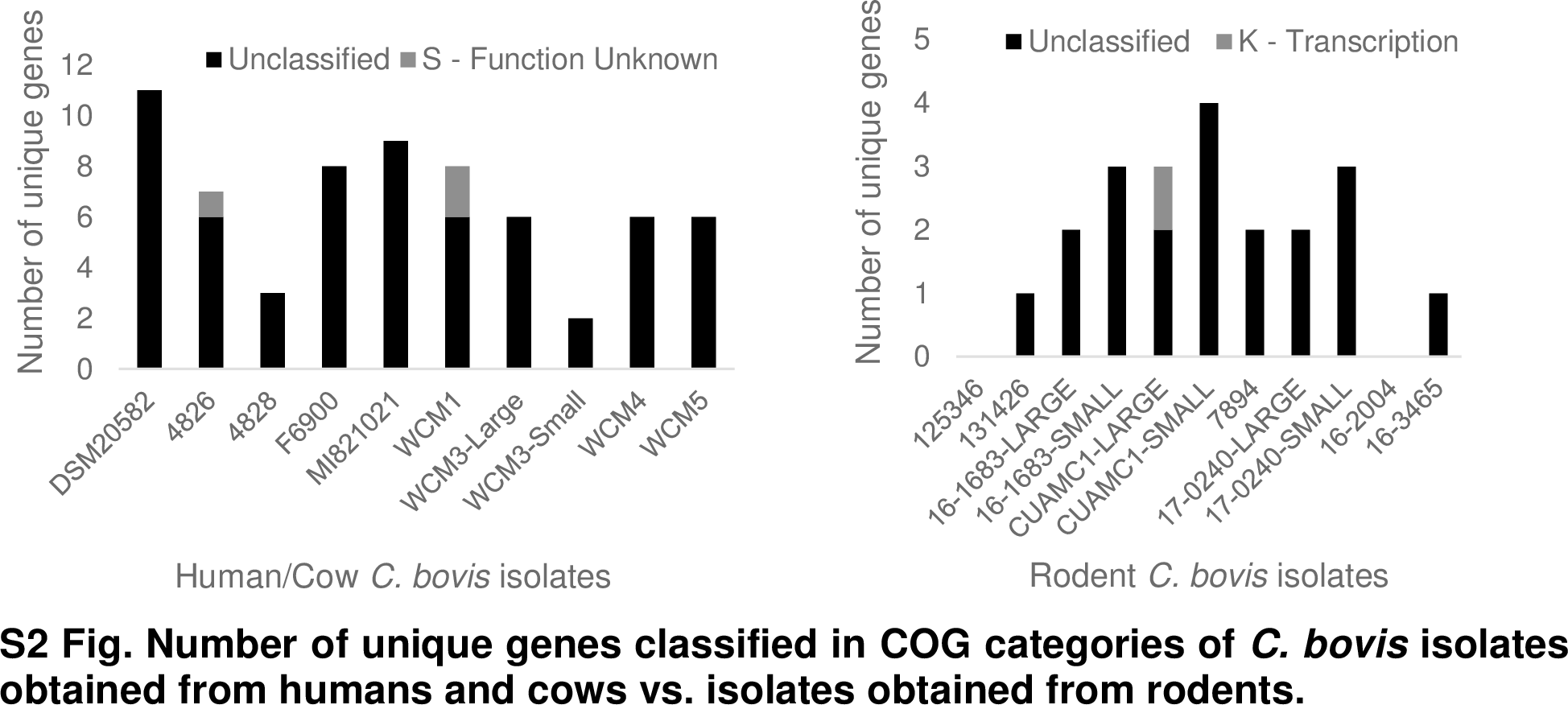

Supplement: S3 Fig — (TIF) [file pone.0209231.s008.tif]
